# Metapangenomics of wild and cultivated banana microbiome reveals a plethora of host-associated protective functions

**DOI:** 10.1186/s40793-023-00493-x

**Published:** 2023-04-21

**Authors:** Simrandeep Singh, Shiva A. Aghdam, Rachel M. Lahowetz, Amanda M. V. Brown

**Affiliations:** 1 Department of Microbiology, University of Illinois, Urbana, IL USA; 2 Department of Biological Sciences, Texas Tech University, Lubbock, TX USA; 3 Department of Molecular Virology and Microbiology, Baylor College of Medicine, Houston, TX USA

**Keywords:** Disease protection, Endophytic bacteria, Metagenomics, Endosphere, *Musa balbisiana*, Banana, Pandemic disease, Microbiome, Diversity, Metapangenomics

## Abstract

**Background:**

Microbiomes are critical to plants, promoting growth, elevating stress tolerance, and expanding the plant’s metabolic repertoire with novel defense pathways. However, generally microbiomes within plant tissues, which intimately interact with their hosts, remain poorly characterized. These endospheres have become a focus in banana (*Musa* spp.)—an important plant for study of microbiome-based disease protection. Banana is important to global food security, while also being critically threatened by pandemic diseases. Domestication and clonal propagation are thought to have depleted protective microbiomes, whereas wild relatives may hold promise for new microbiome-based biological controls. The goal was to compare metapangenomes enriched from 7 *Musa* genotypes, including wild and cultivated varieties grown in sympatry, to assess the host associations with root and leaf endosphere functional profiles.

**Results:**

Density gradients successfully generated culture-free microbial enrichment, dominated by bacteria, with all together 24,325 species or strains distinguished, and 1.7 million metagenomic scaffolds harboring 559,108 predicted gene clusters. About 20% of sequence reads did not match any taxon databases and ~ 62% of gene clusters could not be annotated to function. Most taxa and gene clusters were unshared between *Musa* genotypes. Root and corm tissues had significantly richer endosphere communities that were significantly different from leaf communities. *Agrobacterium* and *Rhizobium* were the most abundant in all samples while Chitinophagia and Actinomycetia were more abundant in roots and Flavobacteria in leaves. At the bacterial strain level, there were > 2000 taxa unique to each of *M. acuminata* (AAA genotype) and *M. balbisiana* (B-genotype), with the latter ‘wild’ relatives having richer taxa and functions. Gene ontology functional enrichment showed core beneficial functions aligned with those of other plants but also many specialized prospective beneficial functions not reported previously. Some gene clusters with plant-protective functions showed signatures of phylosymbiosis, suggesting long-standing associations or heritable microbiomes in *Musa*.

**Conclusions:**

Metapangenomics revealed key taxa and protective functions that appeared to be driven by genotype, perhaps contributing to host resistance differences. The recovery of rich novel taxa and gene clusters provides a baseline dataset for future experiments *in planta* or in vivo bacterization or engineering of wild host endophytes.

**Supplementary Information:**

The online version contains supplementary material available at 10.1186/s40793-023-00493-x.

## Background

Endophytic microbiomes (i.e., the endosphere) appear to contribute significantly to plant health through promoting growth, stress tolerance, and disease resistance, but their functions remain poorly understood compared to soil and rhizosphere microbiomes [1, 2, 12, 24, 39, 57, 66, 87]. Endosphere microbes can be difficult to study because most species cannot be cultured [88, 105]. However, increasingly, culture-free approaches of whole endophyte communities *in planta* have illuminated some key features of these systems. For example, early work suggested endosphere taxonomic profiles are largely conserved within species, although they can vary with environment, host tissue, and host genotype [15, 60, 91]. Other studies confirm these patterns and show some degree of heritability of endosphere members [15, 34, 106, 109]. Given these findings, one unanswered question is how plant domestication and practices such a clonal propagation affect the endosphere: do these processes deplete disease protective endosphere-based functions?

To address this gap, the present study investigates endosphere diversity and functional variance among varieties of wild and cultivated banana and plantain (*Musa* spp.). Banana is an important plant for studying the effects of domestication and clonal propagation on the endosphere, with many susceptible and resistant cultivars and ‘crop wild relatives’ (CWR) that may hold promise for developing microbe-based disease protective inocula [51, 58, 100, 111]. Banana is also important as a major global food commodity, having high production volume and trade value [31] (https://www.fao.org/markets-and-trade/commodities/bananas/en/). Due to its triploid clonal propagation and large monoculture production, major commercial cultivars such as *Cavendish* bananas are susceptible to pandemic *Fusarium* wilt (Panama disease) tropical race 4 (*Foc* TR4) [16, 19, 44, 50, 58, 68, 78–80, 111]. It remains unclear if some of the disease resistance variance among *Musa* cultivars, such as wild diploid genotypes derive from differences in endosphere composition. Yet, to date, the taxonomic profiles and basic functions of root and leaf endospheres across wild and cultivated *Musa* spp. are largely unknown.

To investigate potential host genotype and host tissue derived effects on *Musa* spp. endospheres, we compared root and leaf endospheres from *Musa* genotypes grown in sympatry. By sampling plants grown close together, under the same conditions on a small farm, we assume a shared pool of microbiota available to colonize the endospheres of these plants. Differences in endosphere profile, therefore, should largely reflect differences in host response to, or control of colonizing microbes. Our approach focused on shotgun metagenomic sequencing. To reduce non-target plant DNA and thereby reduce the cost of sequencing [52], we employed a culture-free microbiome enrichment protocol previously shown to be successful and unbiased in soybean [43]. Our analyses centered on metapangenomics, which is a combined analysis of all unique and shared genes and functions in the community. Based on past studies of other plants, we predicted banana root endospheres would be more diverse taxonomically than leaf endospheres, but that key functions would be shared [34, 109] and that wild diploid banana endospheres would be more taxonomically and functionally diverse than that of cultivated triploids. Furthermore, we predicted to see both transient microbes shared across sympatric plants without phylogenetic congruence with their hosts, which we expected would be enriched for pathogenic or opportunistic functions, and microbes unique to hosts genotypes indicative of specialized genotype-specific endosphere colonizers. We predicted the latter group would be enriched for host-beneficial functions.

This study is the first to compare endospheres of root and leaf from wild *Musa* genotypes (*Musa textilis*, *Musa sikkimensis*) and representatives of the *Musa* A- and B-genomes (estimated to have diverged about 5.4 million years ago [38, 107]), including triploid AAA cultivars, diploid wild BB (*Musa balbisiana*), and the hybrid AAB. Results showed that microbial community structure and metapangenome function differed dramatically among tissues and host genotypes, indicating that *Musa* hosts drive recruitment and retention of microbial communities with distinct putative disease protective profiles. Furthermore, our analyses uncovered a substantial wealth of uncharacterized genes and taxa for future study.

## Methods

### Sample collection

To examine microbiomes from a range of banana (*Musa* spp.) genotypes exposed to the same soil and environment, seven different cultivars and species were collected from a small farm (~ 9 ha) in Homestead, Florida in December of 2016: two triploid AAA cultivars that are part of the Giant Cavendish group belonging to *Musa acuminata*, Dwarf Cavendish (denoted DC) and Williams Hybrid (denoted WH), two diploid wild BB plants belonging to *Musa balbisiana*, including a standard variant (denoted MB) and ‘Thai Black’ variant (denoted BB), a hybrid triploid AAB that is a widespread cooking plantain and a cross of *M. acuminata* x *M. balbisiana* genotypes, FHIA-25 (denoted FH), and two other diploid species, *Musa sikkimensis* known as the Darjeeling banana (denoted MS), and *Musa textilis* which is an economically important fiber plant, known as Manila hemp (denoted MT) (see Table 1). Sampled plants consisted of “suckers” (budding small plants ~ 70–90 cm tall) growing from the base of full-sized fruiting banana “trees”. Each plant was cut free from its mother plant with a clean blade that preserved the rhizome (corm) and roots of the sucker. Each small plant was vegetative (not flowering) and had similar corm and root volume, and appeared to be healthy with no disease symptoms. To assess the differences in microbiota present in above-ground and below-ground tissues, two samples were taken for each plant cultivar, one from the leaves (L) and another from the combined root plus corm tissues of the same plant. Although labeled root (R) or corm (C) in sample codes, both notations represent the same set of tissues sampled for each plant, and are hereafter denoted ‘root’ for the total below-ground tissues. After washing with tap water for 10 min, tissues were surface sterilized following standard protocols [84] by immersing in 70% ethanol for 1 min, 2.5% sodium hypochlorite solution for 3 min, 70% ethanol for 1 min, and then rinsing three times with sterile distilled water.

**Table 1:** Sampled genotypes, tissues, and sequence output for 14 *Musa* specimens analyzed in this study

Sample name	Species (Cultivar)	Genotype	Tissue	Sequenced nucleotides (Gbp)	Percentage of nucleotides not mapped to *Musa*
WHL	*M. acuminata* (Williams Hybrid)	AAA	Leaf	0.3	26.51
WHC	*M. acuminata* (Williams Hybrid)	AAA	Root & Corm	0.6	13.3
DCL	*M. acuminata* (Dwarf Cavendish)	AAA	Leaf	10.9	97.72
DCR	*M. acuminata* (Dwarf Cavendish)	AAA	Root & Corm	11.6	63.1
MBL	*M. balbisiana*	BB	Leaf	14.5	93.96
MBR	*M. balbisiana*	BB	Root & Corm	15.9	24.66
BBL	*M. balbisiana* ‘Thai Black’	BB	Leaf	19.8	81.5
BBC	*M. balbisiana* ‘Thai Black’	BB	Root & Corm	19.1	14.02
FHL	*M. acuminata* (FHIA-25)	AAB	Leaf	21.8	77.38
FHC	*M. acuminata* (FHIA-25)	AAB	Root & Corm	17.3	26.26
MSL	*M. sikkimensis*	S-genome	Leaf	4.0	80.4
MSR	*M. sikkimensis*	S-genome	Root & Corm	6.7	66.79
MTL	*M. textilis*	T-genome	Leaf	7.0	79.72
MTC	*M. textilis*	T-genome	Root & Corm	12.0	22.32

Includes sample name, *Musa* species or cultivar, genotype, tissue location, amount of shotgun metagenomic sequence data in giga base pairs sequenced per sample, and percent of sequence data comprising the microbiome (i.e., not mapping to *Musa* spp.)

### Culture-free enrichment for endophytes

To perform shotgun metagenomics on endophytic microbiota while reducing the cost of sequencing non-target plant genomes, a culture-free microbiome enrichment protocol was adopted that was previously shown to be successful in soybean [43]. Specifically, 100 g of each plant sample was homogenized in a sterilized blender with 400 mL of ice-cold bacterial cell extraction ‘BCE’ buffer (50 mM Tris–HCl pH 7.5, 1% Triton X-100, 2 mM 2-mercaptoethanol). All the following steps were performed at 4 °C, including centrifugation. The homogenate was filtered through a sterilized Miracloth (EMD Millipore) rayon-polyester mesh, then centrifuged at 500xg for 5 min and the supernatant was transferred to clean tubes and centrifuged at 5000xg for 20 min. Resulting supernatant was discarded and the pellet was resuspended in 50 mL of BCE, then filtered through a sterilized Kimwipe (Kimberly-Clark). The filtrate was centrifuged at 5000xg for 10 min. The supernatant was discarded, and the pellet was resuspended in 35 mL of BCE, before repeating the Kimwipe filtration. The final pellet was resuspended in 6 mL of 50 mM Tris–HCl (pH 7.5), and each 1 mL aliquot was slowly pipetted over 4 mL of Nycodenz® solution (3.2 g of Nycodenz® + 4 mL of 50 mM Tris–HCl, pH 7.5). All 6 tubes were then centrifuged at 5000xg for 40 min. After centrifugation, a white-to-grey band of microbial cells at the interface of the layers was collected.

### DNA extraction, Illumina library preparation and sequencing

DNA was isolated from the enriched microbiome layer using the QIAGEN DNeasy Blood & Tissue Kit (Valencia, CA) following the manufacturer’s directions. DNA was checked for quantity and quality on the Nanodrop spectrophotometer, then ~ 0.5 to 1 μg of DNA was used for shotgun metagenomic library preparation with the QIAseq FX 96 DNA Library Kit (Valencia, CA) following the manufacturer’s directions. Libraries were checked for quality and quantified on the TapeStation 2200 (Agilent) before normalizing and pooling libraries for sequencing. HiSeq 2500 (Illumina) paired-end sequencing was performed at the Center for Biotechnology and Genomics at Texas Tech University and then at Genewiz, Inc (NJ) with reads of 105 and 151 bp, respectively. Reads from the same sample library were concatenated prior to analysis.

### Host plant phylogenetic analysis

To evaluate plant host relationships, an initial assembly from all reads was performed to enable isolation of marker genes from the plant. First, reads were trimmed and filtered with Trimmomatic v0.38 [14] and merged overlaps were assessed with Pear v0.9.11 [112]. Resulting reads were de novo assembled with metaSPAdes in SPAdes toolkit v3.13.0 [9, 72] with read-error correction and kmer lengths of 21, 33, 45, 59, 73 and 99. Resulting contigs with blastn match to chloroplast/plastid gene *ycf1*, a gene previously shown to be well-suited for resolving plant phylogenies [5, 61, 69], were extracted with custom scripts and aligned using Mafft v1.0.4 [46] or Clustal Omega 1.2.3 [92] along with outgroup *Musa* spp., *Ensete* spp., and *Musella* spp. sequences downloaded from NCBI. Phylogenetic analyses were performed using RAxML v4.0 [96], assessing bootstrap support from 1000 replicates using the ML GTR Gamma nucleotide model, with rate heterogeneity alpha estimated, and with rapid bootstrapping and search for the best-scoring ML tree (-f a -x 1), then Bayesian phylogeny estimation was performed with MrBayes v2.2.4 [42, 86] with the GTR + G model with 4 categories, and Markov chain Monte Carlo settings of: chain length 1,100,000, 4 heated chains, heated chain temp 0.2, subsampling frequency 200, Burn-in length 100,000, with random seed 31,569, and priors with unconstrained branch lengths GammaDir (1,0.1,1,1), checking for convergence with minESS > 200. Final phylogenetic trees were visualized in FigTree v1.4.4 (http://tree.bio.ed.ac.uk/software/) with labels and color added in Adobe Illustrator (Adobe Systems, San Jose, CA).

### Mapping against host genomes

Illumina paired-end read overlaps were merged using Pear, then merged and paired non-overlapping reads were mapped to *Musa* genomes to remove residual plant DNA not fully removed by the Nycodenz enrichment. Reads were mapped to two *Musa* spp.: the wild type *M. balbisiana* (2n = 22) PKW PacBio assembly (NCBI BioProject PRJNA432894 [107]), and the *M. acuminata* subsp. *malaccensis* (2n = 22) Sanger, Roche/454 GSFLX and Illumina GAIIx assembly (NCBI BioProject PRJEA82777 [27]) using BWA-MEM in BWA v0.7.17 [53]. To avoid mapping to potentially unidentified endophytic DNA that might be present in the smaller unplaced scaffolds of the reference assembly, 87.27% of the *M. balbisiana* assembly and 70% of the *M. acuminata* assembly were anchored to the 11 *Musa* linkage groups and only these were used as a reference.

### Quality filtering, metagenome assembly, and initial taxonomic assignments

Trimmomatic was used to trim and filter merged and paired reads before assembly with metaSPAdes in SPAdes toolkit v3.13.0 [9, 72] with read-error correction and using kmer lengths of 21, 33, 45, 59, 73 and 99. Assembly statistics were calculated using QUAST v5.0.2 [37]. Initial assignment of scaffolds to taxa (i.e. taxonomic binning) was performed by searching against the NCBI nr database (accessed Dec 2020) using DIAMOND v2.0.9 [17] with the sensitive flag enabled, minimum e-value threshold of 1e-05, block size of 16 and single chunk used for processing the seed index. Reads were mapped to these scaffolds using BWA-MEM to assign reads to the corresponding taxa resulting from DIAMOND blast.

### Diversity analysis based on binned, clustered reads

Differences in microbial community richness across variables such as plant tissue and host genotype were analyzed using the phyloseq package in R [62], through processed read data clustered into Operational Taxonomic Units (OTUs) with the taxonomic assignment of the OTUs to species level. Species richness curves for all samples were generated based on non-normalized read counts with rarefaction curves generated from random subsampling of up to 5 million reads from the original OTU table. Microbial species richness indices (i.e., Chao, ACE, Shannon, and Simpson indices) were calculated based on the OTU data normalized to the median of the sample read counts between samples with highest and lowest read counts. Principal component analysis (PCoA) and non-metric multidimensional scaling (NMDS) were also performed based on Bray–Curtis dissimilarity as a factor of beta diversity across samples to account for the difference in OTU abundance normalized to the median of the sample read counts. Permutational Multivariate Analysis of Variance (PERMANOVA) was performed to assess the relationship between differences in OTU abundance in the microbial communities of samples as a function of genotype and location of tissue using ‘Adonis’ in the Vegan package [29] in R.

Abundance of individual taxa was compared in samples grouped according to genotype and plant tissue by ANOVA and Welch’s two sample *t*-test, respectively, in R (Vegan package). All comparisons between samples were based on normalized values, unless stated otherwise. For genotype comparisons between groups, ANOVA tests were followed by pairwise Welch’s two sample *t*-tests.

### Core and unique endophytic microbiomes

To evaluate taxon differences, core microbiomes and unique and shared microbiomes, down to the strain-level, lowest annotated taxa resulting from DIAMOND blast searches were compared independently, after normalizing scaffolds from a coverage-based ordered list such that the same proportion of each root assembly was kept; thus, we removed lower coverage scaffolds in inverse proportion to the relative number of reads. Shared taxa were evaluated and displayed using the online Venn drawing tool http://bioinformatics.psb.ugent.be/webtools/Venn/ and the proportional Venn tool nVenn [77].

### Metagenomic repertoire comparisons and gene ontology enrichment analysis

To evaluate microbiome pangenome (or ‘metapangenome’) and core (shared) gene repertoires, total metagenome assemblies resulting from normalizing as described above (according to relative scaffold read coverage among samples) were annotated with Prokka v.1.14.6 [90]. Ortholog clusters were assessed using Roary v3.13.0 [75] on gff outputs from Prokka, with parameters -e for codon-aware alignment in PRANK [59] and -i 60 to detect distant orthologs. Output gene presence or absence clusters were depicted with the online Venn drawing tools described above, using the full set of genes, annotated with the inclusion of genes of unknown function annotated as ‘hypothetical protein’ and forming clusters denoted as separate ‘groups’. We also analyzed the metapangenome patterns for genes at a higher functional level by consolidating diverged variants of genes (e.g., combining separately clustered genes of the same name) and removing gene clusters with no known function.

Gene ontology (GO) enrichment was assessed for various overlapping and non-overlapping gene homologous gene sets resulting from Roary gene clustering and metapangenome analysis. Initial gene lists from Roary contained large numbers of redundant clusters with the same gene name (e.g., of the form adhP_1, adhP_2, adhP_3 etc.). Although these gene variants may in some cases serve slightly different functions, it was not possible to annotate this for the bulk of the data, therefore, such redundant clusters were combined to single gene names for the purpose of GO enrichment analysis. Gene clusters listed as ‘group_#’, with no functional annotation were also removed. To create a master list of curated GO ID-to-gene mappings, GO ID-gene lists were downloaded from the UniProtKB database for 72 of the most abundant genera in the banana microbiomes, spanning most major clades of bacteria found in the samples. Any missing genes or synonyms were resolved by cross-referencing with MetaCyc [21] and KEGG [45] databases. This master GO ID-gene mapping list was used in enrichment analysis in topGO v2.4.0 [4]. This software assesses the GO-term graph topology with ‘weight01.fisher’ which returns multiple testing independent p-values. An R script aip_topgo_usage.consider_universe.R (https://github.com/lyijin/topGO_pipeline/) was used to rapidly process topGO analyses. GO term semantic similarity plots for biological processes were depicted using the python package ‘GO-Figure!’ [85] with transparent overlays of plots, for comparison, prepared in Adobe Illustrator.

To assess potential congruence between endophytic microbiome gene clusters and host banana plants, we performed phylogenetic reconstruction on all gene clusters using FastTree 2.1.8 [81] with the generalized time-reversible model and other parameters set to the default, then extracted newick-format branch topologies for analysis of approximate phylosymbiosis to the host plant phylogeny based on topology of closest clusters of two and three host genotypes matching microbial gene ortholog clusters. Resulting approximately phylosymbiotic ortholog clusters were then analyzed for functional enrichment compared to the set of all microbial genes in topGO, and the taxonomic bin corresponding to this gene set was analyzed using DIAMOND blast, with host-to-microbial strain-to-gene associations plotted in a Sankey drawing using the sankeyNetwork tool in networkD3 in R.

## Results

### Endophytic microbiome enrichment success and metagenome assembly quality

Sequencing of 14 *Musa* spp. samples (Table 1) generated 161.5 Gbp of raw sequence data. Initial output (Additional file 1: Table S1) showed similar values for reads mapping to the two reference *Musa* species except for 4 leaf samples, of which most showed higher mapping to *M. acuminata* even though the source plant sample was from were from *M. balbisiana*, suggesting possible endophyte contamination in this reference assembly. Based on these results and the *M. balbisiana* reference quality (with several-fold higher N50 values of the compared to the *M. acuminata* reference, with no gaps between scaffolds reads), we chose to use only the *M. balbisiana* reference for plant DNA removal using Samtools v1.9 [54] prior to downstream analysis. Alignment showed a higher percentage of reads aligned to the reference *M. balbisiana* genome in above-ground samples as compared to below-ground samples (Additional file 1: Fig. S1A, B) (Welch’s two sample *t*-test, *P* = 0.0002). After mapping and filtering 45.3 Gbp of sequenced data remained for assembly of endophytic microbiomes (Additional file 1: Fig. S1C, D), which resulted in about 2.2 million scaffolds with total length of 2.03 Gbp, an average N50 of 2191 bp, with over 11,000 scaffolds greater than 10 kbp long and 1250 scaffolds greater than 50 kbp (Additional file 1: Table S2). According to rarefaction curves (Additional file 1: Fig. S2) samples WHL, WHC, and DCL were undersampled (had insufficient read coverage to represent taxonomic diversity) and thus our statistical comparisons of differentially abundant taxa (below) excluded these samples.

### Phylogenetic relationships among sampled banana cultivars

**Fig. 1:**
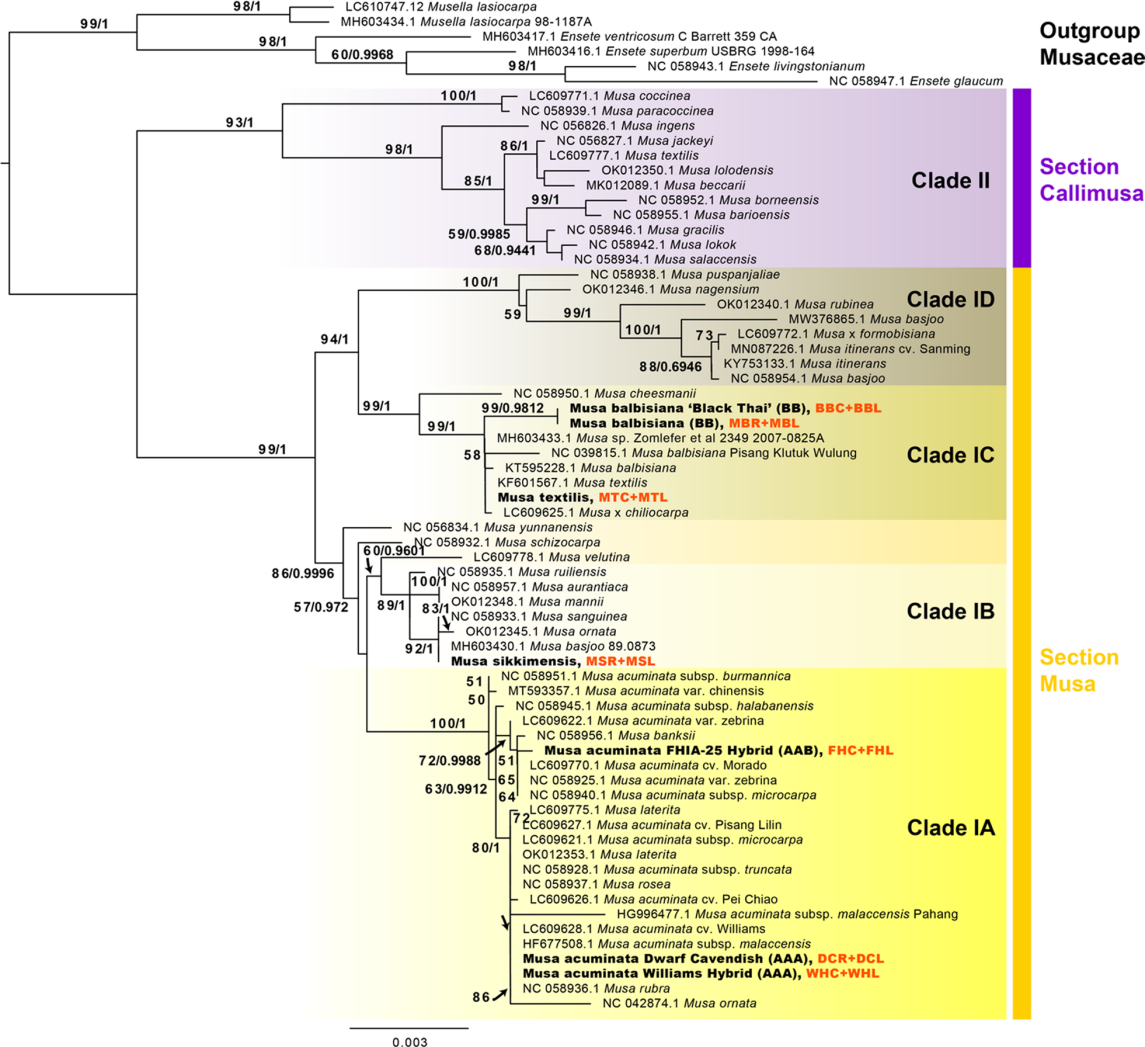
Phylogeny of *Musa* samples collected in this study, based on the chloroplast gene *ycf1*, generated from 6814 aligned nucleotide positions. Outgroup and ingroup sequences were derived from NCBI GenBank, with accession numbers shown by taxa. Phylogenetic tree reconstruction was performed in RAxML with the GTR + Gamma model, with support from 1000 bootstrap replicates shown on branches, next to posterior probabilities for supported nodes obtained from analysis with MrBayes from Bayesian 50% majority rule with GTR + G with 4 rate categories model. Samples from this study are shown in bold font

The *ycf1* plastid gene sequences were identical between root and leaf within each sampled genotype, as expected, and differed significantly between plant cultivars or species sufficiently for phylogenetic analysis. Phylogenetic analyses from the *ycf1* plastid gene using an alignment block of 6814 bp positions produced consistent results in both ML and Bayesian analyses, which strongly supported separate clades for genera *Musella*, *Ensete*, and *Musa*, and supported clades corresponding to sections Callimusa (Clade II) and Musa (Clade I) (Fig. 1). Within section Musa, our sampled specimens fell within separate supported clades. Our samples BB and MB (*M. balbisiana*) had high (99.8%) *ycf1* gene similarity and clustered closely in a clade dominated by other BB genotypes (Clade ID). Our sample MT (*M. textilis*) was similar in *ycf1* gene sequence (98.9%) to the MB and BB, and clustered within the same clade, along with other *M. textilis* and *Musa* x *chiliocarpa* sequences. Sample MS (*M. sikkimensis*) was less similar in *ycf1* sequence to MB (94.6% identity), and clustered in distinct clade (Clade IB) along with *M. basjoo*, *M. ornata*, and *M. sanguinea* (Clade IC). Sample FH (FHIA-25 hybrid) clustered in a separate clade with *M. banksii* and several *M. acuminata* cultivars including cv. morado, var. zebrina, and subsp. *macrocarpa*, with 95.8% similarity to MB in the *ycf1* gene. Samples DC (Dwarf Cavendish) and WC (Williams Hybrid) were ~ 98.8% similar in *ycf1* gene sequence, ~ 95.8% similar to MB, and clustered with *M. acuminata* cv. Williams, subsp. *malaccensis*, and numerous other cultivars of *M. acuminata* (Clade IA) (Fig. 1).

### Shared and unique microbial strains from banana endophytic microbiome communities

Detailed analyses of taxa at the lowest taxonomic levels (i.e., mostly annotated to species- or strain-levels, and hereafter denoted ‘species or strains’) uncovered sets of shared taxa (i.e., ‘core microbiomes’), partly shared taxa (i.e., ‘shell microbiomes’), and unshared taxa with all together 24,325 distinct taxa including 96.76% bacteria, 364 fungi from 218 genera, 235 viruses, and 186 archaea (Additional file 2: Table S3). Note that for these analyses, WH samples were excluded due to undersampling, leaving 12 samples, which were normalized as described in the methods. Among these samples there were just 185 universally shared or ‘core’ species or strains among all root and leaf samples (Additional file 1: Fig. S3A), comprising 184 bacteria from 56 genera, consisting of mostly environmental and phytopathogenic species or strains and one universally shared endogenous dsDNA Badnavirus, Banana streak virus (BSV). There were 15,115 species or strains (62.13% of taxa) occurring in two or more samples, i.e., ‘shell’ taxa (Additional file 1: Fig. S3A), and 9025 species or strains (37.1% of taxa) occurring in only one tissue sampled, i.e., ‘cloud’ taxa (Additional file 1: Fig. S3A). Pan-community comparisons between all taxa from root and all taxa from leaf samples showed most taxa were unique to roots (16,360 taxa or 62.25% of taxa) whereas few taxa were unique to leaf tissues (690 taxa or 2.84%). The number of unshared ‘cloud’ taxa varied among samples (MBR = 2822, BBC = 2297, DCR = 1988, FHC = 447, MTC = 431, MSR = 414, BBL = 192, FHL = 115, MSL = 98, MTL = 96, MBL = 93, DCL = 32) (Additional file 1: Fig. S3A, Additional file 2: Table S3). Because ‘core’ universally shared taxa among samples was impacted by the low diversity of taxa in leaf tissues, we also compared pan-community overlap after combining leaf and root taxon lists for each *Musa* genotype (Additional file 1: Fig. S3B), which showed 2992 taxa shared among pan-communities of all genotypes, with > 2000 taxa unique to each of MB, BB, and DC genotypes. Pan-community analysis comparing the samples with the largest numbers of unique ‘cloud’ taxa, MBR, BBC, and DCR (which comprise BB and AAA genotypes), showed 5540 taxa (30%) unique to the BB genotype root (MBR/BBC) compared to 3758 taxa (20.3%) unique to AAA genotype root (DCR) (Additional file 3: Table S4).

### Taxonomic composition and bacterial predominance in banana endophytic microbiomes

**Fig. 2:**
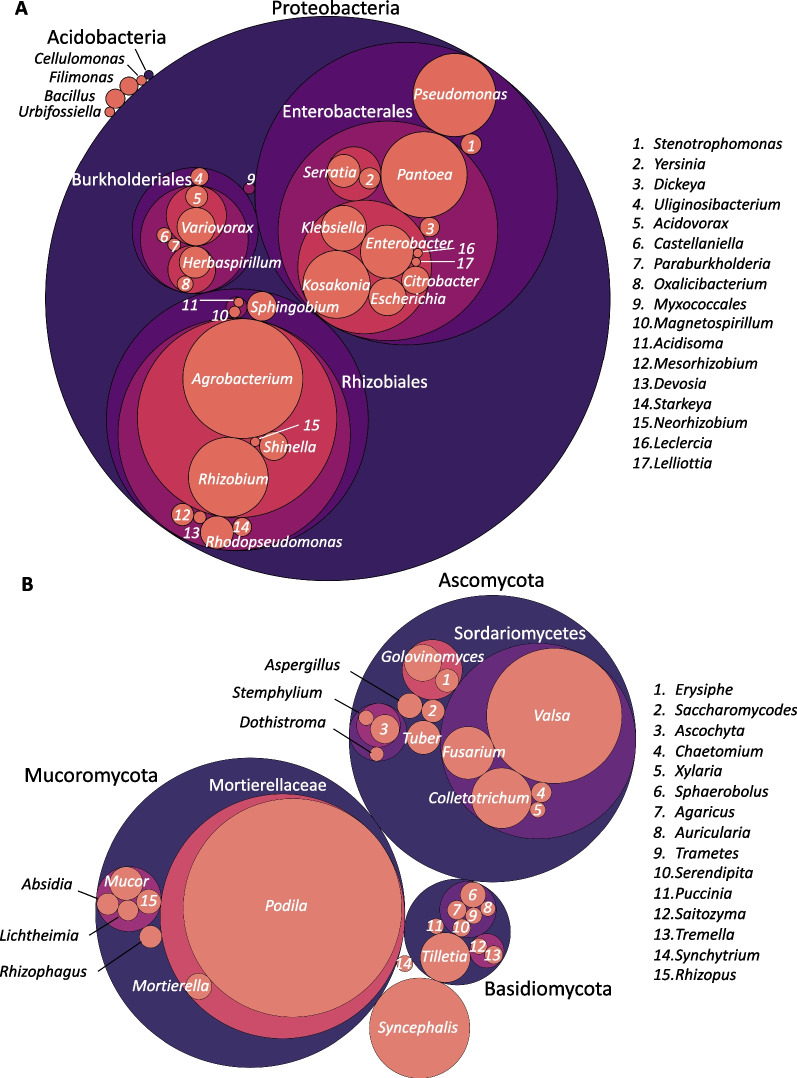
Taxonomic classification of bacteria (**A**) and fungi (**B**) from endophytic microbiomes of all *Musa* samples (Table 1) (non-normalized), where circle area is proportional to taxon abundance at various taxonomic levels, based on reads mapped to taxonomically classified scaffolds based on DIAMOND blastn to the nr database

Comparisons of abundance based on reads mapped to contigs revealed higher-level taxonomy across all samples was dominated by Bacteria (66.65% of reads) followed by Eukaryota (12.73% of reads) with just 0.08% of reads matching Viruses, and 0.01% of reads matching Archaea. Many reads (20.52%) remained unclassified to any taxon (Additional file 1: Table S5). Of the 79.48% reads matched to the nr database, more than 94% were successfully identified to the genus level. Within eukaryotes, 16% of reads matched Fungi, 78.1% matched Viridiplantae, and 5.5% matched Metazoa. Viridiplanta and Metazoa reads and contigs were filtered out before further downstream analyses. Higher-level microbial taxa composition varied between samples, with bacterial reads being rarest (< 2%) in leaf samples of Dwarf Cavendish and up to 85% in other samples (Additional file 1: Fig. S1). In total read counts (non-normalized across samples) at the phylum level, Proteobacteria and Bacteroidetes dominated the endophytic bacterial communities, with long bacterial scaffolds (> 5 kbp) mostly matching orders Rhizobiales, Enterobacterales, Pseudomonadales, Burkholderiales, Xanthomonadales, and Sphingomonadales (Fig. 2 and Additional file 1: Fig. S4). At lower taxonomic levels, Enterobacterales matched *Enterobacteriaceae* (*Kosakonia, Enterobacter, Klebsiella*, and *Citrobacter*) and *Erwiniaceae* (*Pantoea*), Burkholderiales matched *Comamonadaceae* (*Variovorax* and *Acidovorax*) and Burkholderiaceae (*Paraburkholderia*), whereas Rhizobiales, Pseudomonadales, Xanthomonadales, and Sphingomonadales comprised mostly *Rhizobiaceae* (*Agrobacterium* and *Rhizobium*), *Pseudomonadaceae* (*Pseudomonas*), *Xanthomonadaceae* (*Strenotrophomonas*), and *Sphingomonadaceae* (*Sphingobium*) (Fig. 2 and Additional file 1: Fig. S4). Most fungal reads matched phylum Ascomycota (98.8%), but most of these were *Beauveria bassiana* (> 97%) which is an entomopathogenic insecticide applied in biocontrol, thus, this taxon was removed from further analyses. Remaining fungal reads matched Mucoromycota (0.83%), Zoopagomycota (0.16%) and Basidiomycota (0.13%), with dominant fungal genera including *Podila* (syn. *Mortierella*), *Valsa*, *Syncephalis*, *Colletotrichum*, *Fusarium*, *Tilletia*, and *Golvinomyces* (Fig. 2). *Fusarium* species matches present at trace levels included *Fusarium oxysporum* f. sp. *cubense* in 3 samples: root tissues of *M. balbisiana* ‘Thai Black’ and root tissues of *M. balbisiana* and Dwarf Cavendish.

**Fig. 3:**
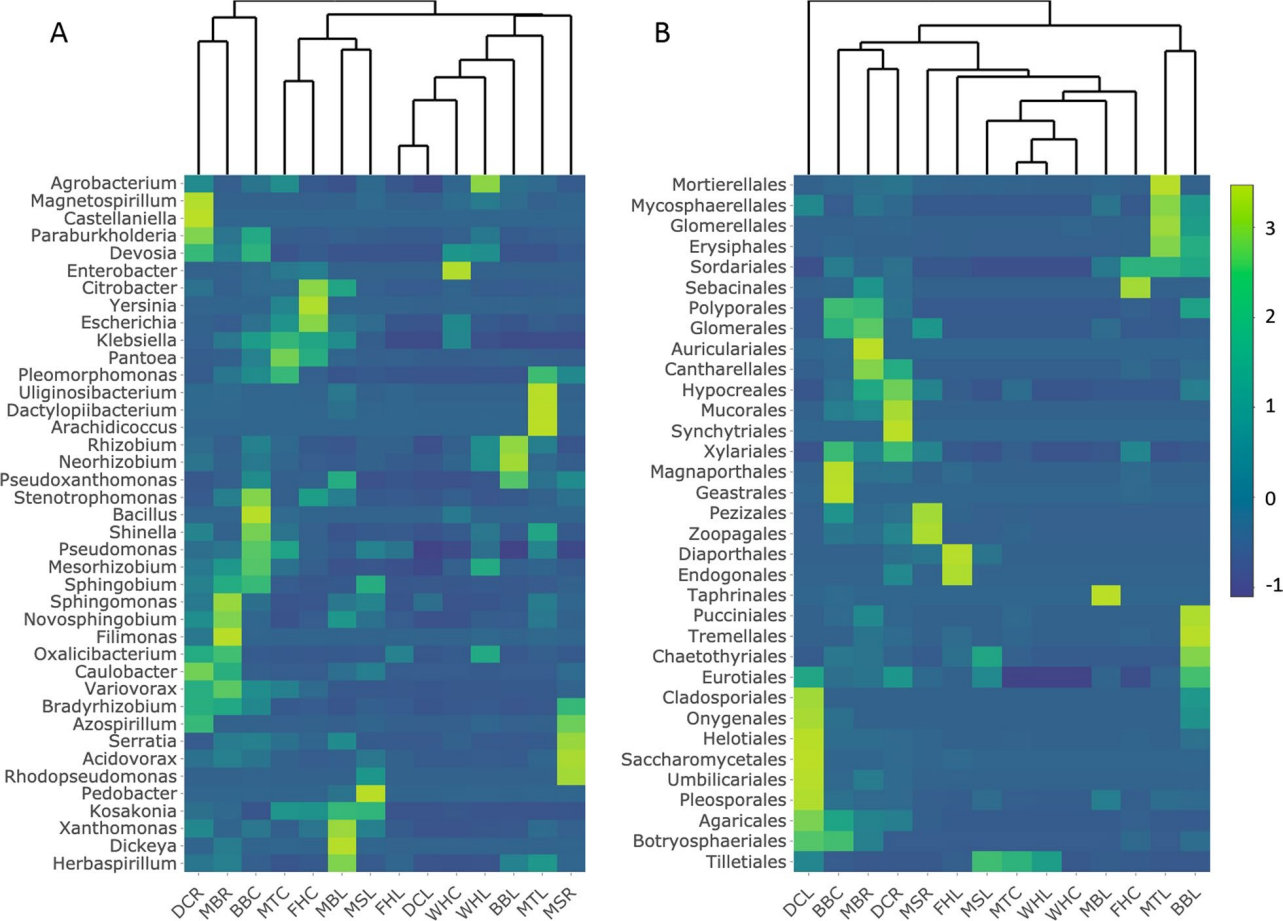
Comparison of dominant bacterial genera (**A**) and fungal classes (**B**) from endophytic microbiomes of *Musa* samples listed in Table 1, based on read abundance mapped to assembled scaffolds showing only taxa occurring at more than 1% in at least one sample. Colored shading on heatmaps indicates relative read abundance of that taxon, normalized for number of microbial reads per sample

After normalizing for relative numbers of reads sequenced among samples, proportions of the most abundant bacteria and fungi varied among samples (Fig. 3). *Rhizobiaceae* was the most abundant bacterial family in all samples except for WHC, FHC and MBL in which *Enterobacteraceae* was most abundant. Below-ground tissues had more Gammaproteobacteria, Chitinophagia, and Actinomycetia compared to above-ground tissues, while above-ground tissues harbored high abundances of Flavobacteria (Fig. 3). Statistical tests of abundance differences showed at higher taxonomic levels that below-ground tissues had significantly more reads assigned to orders Corynebacteriales (Welch’s two sample *t*-test, *p* = 0.01), Hyphomonadales (Welch’s two sample *t*-test, *p* = 0.04) and Rhodospirillales (Welch’s two sample *t*-test, *p* = 0.04). Several members of Hyphomicrobiales, Burkholderiales and Xanthomonadales were also significantly more abundant in below-ground tissues (Additional file 1: Table S6). Three members of Gammaproteobacteria, *Moraxellaceae* (*p* = 0.002, ANOVA), *Luteimonas* (*p* = 0.005) and *Solimonas* (*p* = 0.04) were significantly more abundant in the BB *Musa* genotype whereas Alphaprotebacteria such as Rhizobiales (*p* = 0.005, Welch’s two sample t-test), *Hoeflea* (*p* = 0.03) and *Neorhizobium* (*p* < 0.05) were significantly more abundant in *M. sikkimensis* and *M. textilis* compared to FHIA-25 (AAB) (Additional file 1: Table S7).

Many samples contained sequences matching species of Bacteroidetes and Gammaproteobacteria that were previously observed as targets of interest for controlling Panama disease (potentially inhibiting *Fusarium oxysporum* f. sp. *cubense* activity) in varying levels among samples, from genera such as *Chitinophaga*, *Chryseobacterium*, *Filimonas*, *Citrobacter*, *Stenotrophomonas*, *Enterobacter*, *Klebsiella*, *Kosakonia*, and some *Pseudomonas* (Additional file 1: Fig. S5).

### Richness and diversity of banana endophytic microbiomes

**Fig. 4:**
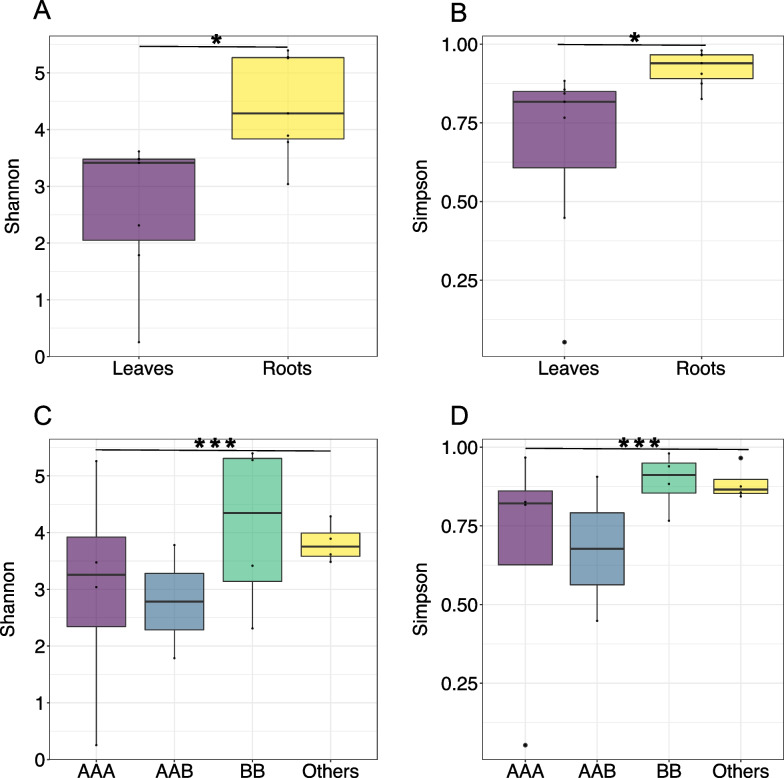
Comparison of alpha diversity in endophytic microbiomes of 14 sampled *Musa* cultivars and tissues grouped according to tissue location (leaves or roots) (**A**, **B**) or genotype (*M. acuminata* Dwarf Cavendish and Williams Hybrid = AAA, *M. acuminata* FHIA-25 = AAB, *M. balbisiana* and *M. balbisiana* ‘Thai Black’ = BB, *M. sikkimensis* and *M. textilis* = Others) (**C**, **D**). Diversity was calculated by either the Shannon-index or Simpson-index based on reads mapped to taxonomically classified scaffolds, statistically significant differences shown with asterisks for *Welch’s two sample t-test or (**A**: *p* = 0.01 **B**: *p* = 0.02) and ***ANOVA (**C**: A: *p* = 0.06 **D**: *p* = 0.05)

Based on OTUs determined in phyloseq (comprising 22,220 bacterial OTUs and 493 fungal OTUs), analyses showed that root tissues had higher alpha diversity than leaf tissues (Fig. 4) (Additional file 1: Fig. S6) (Welch’s two sample *t*-test Shannon (*p* = 0.01), Chao (*p* = 0.01), ACE (*p* < 0.005) and Observed sequence variants indices (*p* = 0.01)), with Shannon diversity indices ranging from 0.25 (DCL) to 3.61 (MTL) in leaves to 3.03 (WHC) to 5.39 (BBC) in root, with averages of 2.62 ± 0.18 SE and 4.42 ± 0.13 SE, respectively (Additional file 1: Table S8). The highest Shannon diversity indices were observed for samples with the BB genotype (*M. balbisiana* and *M. balbisiana* ‘Thai Black’) (4.1 ± 0.37 SE) and the lowest diversity was observed for AAB genotype samples (2.78 ± 0.7 SE), however, the difference was not significant in one-way ANOVAs among all genotypes (Shannon (*p* = 0.6), Simpson (*p* = 0.5), Chao (*p* = 0.53), ACE (*p* = 0.7)).

**Fig. 5:**
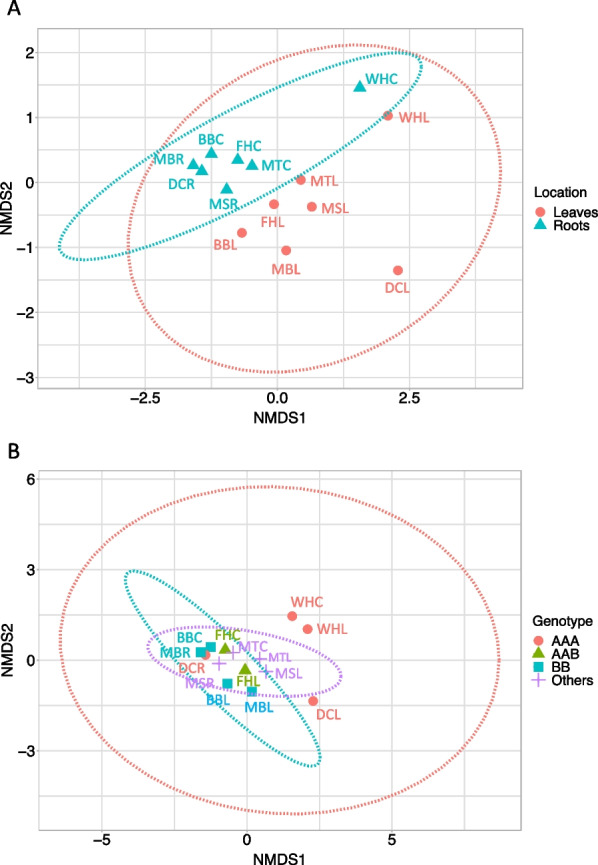
Bray–Curtis dissimilarity among endophytic microbiomes from *Musa* samples listed in Table 1, for tissue location (leaves or roots) (**A**) or plant genotype (*M. acuminata* Dwarf Cavendish and Williams Hybrid = AAA, *M. acuminata* FHIA-25 = AAB, *M. balbisiana* and *M. balbisiana* ‘Thai Black’ = BB, *M. sikkimensis* and *M. textilis* = Others) (**B**). Axes depict first and second dimensions from non-metric multidimensional scaling (NMDS) based on reads mapped to taxonomically classified scaffolds. Ellipses depict 95% confidence intervals fitted to the spatial ordination. Letters by data points refer to *Musa* samples listed in Table 1. Adonis test was significant, *p* = 0.01, for location (**A**), but not for genotype (**B**) *p* = 0.97

Microbiome Bray–Curtis dissimilarity (beta diversity) showed significant differences between above-ground and below-ground tissues (Adonis test, *p* = 0.01), with tighter clustering among below-ground samples in PCoA and NMDS analyses (Fig. 5) (Additional file 1: Table S9). Microbial community profiles overlapped between *Musa* genotypes, with the least scattering observed in *M. sikkimensis* and *M. textilis*, followed by BB and AAB genotypes, whereas microbiomes from the AAA genotype showed the most scattering, but differences between microbiomes from different *Musa* genotypes were not significant (Adonis test, *p* = 0.97). Many OTUs predicted in phyloseq were not shared between samples or were shared among subsets of samples (Additional file 1: Fig. S7) (see more detailed analysis below).

### Metapangenome and core metagenome richness among banana endophytic microbiomes

Metapangenome analyses, which included all annotated genes, taxonomically binned or not, revealed the number of predicted genes per assembled banana endophytic microbiome ranging from 8792 (sample WHL) to 442,820 (sample BBC) with a large portion of genes having no match to genes of known function, with the average percent of genes annotated as “hypothetical protein” being 62%. Because WH samples were undersampled, they were omitted from further metapangenome analysis and assembled scaffolds were normalized as described in the methods. Output genes annotated in Prokka and clustered into homologs in Roary showed 559,108 distinct gene clusters, with more gene clusters that were unique to root (371,805 gene clusters, or 66.5%) compared to the number that were unique to leaf tissue (95,276 clusters, or 17%), and fewer were shared among root and leaf (92,027 clusters). This pattern of richer gene repertoire in microbiomes from roots than leaves was observed for all *Musa* genotypes, and for the set of genes with known function (i.e., removing gene clusters annotated only as hypothetical proteins) consolidating variants with the same gene name or symbol (Additional file 1: Table S10).

**Fig. 6:**
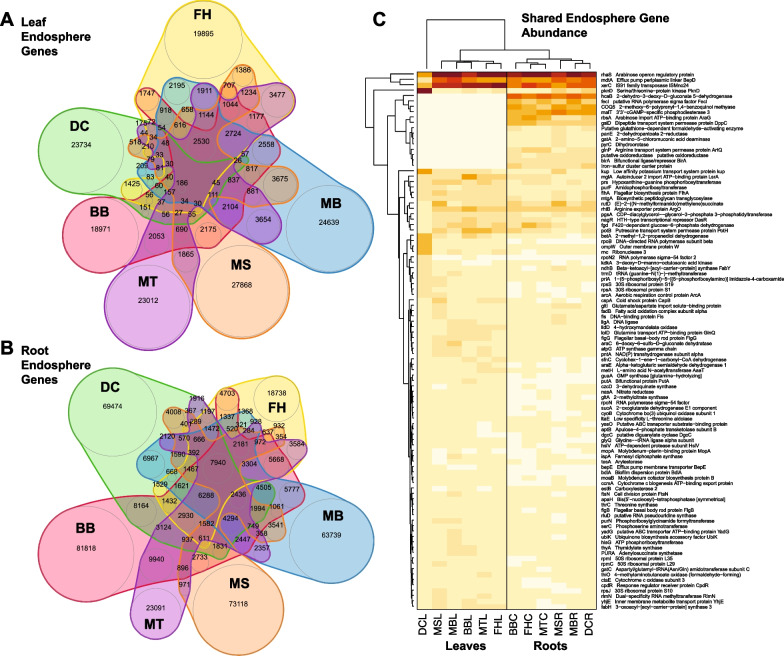
Number of shared and unshared predicted endosphere gene clusters, many of which are annotated only as “hypothetical protein” from endophytic microbiomes from *Musa* samples listed in Table 1, with proportional Venn diagrams (**A**, **B**) shown for leaf microbiome genes (**A**) and root microbiome genes (**B**), after normalizing scaffolds among samples, and relative abundance heatmap for the top 100 most abundant shared genes (**C**) with darkest shading representing highest relative number of copies of each gene. BB = *M. balbisiana* ‘Thai Black’, DC = *M. acuminata* Dwarf Cavendish, FH = *M. acuminata* (FHIA-25), MB = *M. balbisiana*, MS = *M. sikkimensis*, MT = *M. textilis*. Details of gene annotations are shown in Additional file 1: Table S10 and a complete list of gene names is given in Additional file 4: Table S11

Comparisons of root microbiome gene homologs among banana genotypes (Fig. 6A) (Additional file 4: Table S11) showed the majority of genes were not shared between banana samples, with only 7857 gene clusters (1.4%) being “core” genes (universally shared among samples), 125,675 gene clusters (22.48%) being “shell” genes (present in 2 or more but not all samples), and 330,301 gene clusters (59.1%) being “cloud” genes (present in only 1 sample), with BB having the greatest number of cloud genes and FH having the least. This pattern of large number of cloud genes with limited shared gene clusters was more pronounced in leaf than root microbiomes with leaf communities only having 186 core gene clusters, 48,846 shell gene clusters, and 138,274 cloud gene clusters (Fig. 6B), where genotype BB had the fewest cloud genes, while MS had the most cloud genes. Gene clusters that were restricted to root or leaf (i.e., not shared among tissue types) showed a similar pattern across all samples, except that DC had notably fewer genes shared between root and leaf. Comparisons limited to sets of genes with known function (i.e., removing gene clusters annotated only as hypothetical proteins) and consolidating variants with the same gene name or symbol (see Additional file 1: Table S10) showed a greater portion of genes shared in the universal ‘core’ for root (4644 clusters, or 50%) and leaf (726 clusters, or 10.3%) and fewer ‘cloud’ genes in root (1391 clusters, or 14.98%) and leaf (1034 clusters, or 14.72%). Similarly, in this comparison, fewer clusters were found to be unique to leaf (137, or 1.45%) or to root (2400, or 25.5%) compared to the number of clusters shared between these (6886). Despite this more conservative, higher level functional comparison, we still found there were distinct gene clusters between genotypes that followed a similar pattern among samples to analyses described above considering all gene variants. The most abundant (high copy) genes across hosts and tissues included *rhaS* (encoding arabinose operon regulatory protein), *mdtA* (encoding efflux pump periplasmic linker BepD), and *xerC* (IS91 family transposase ISMno24) (Fig. 6C). The most abundant genes shared among roots but not leaves included *hcaB* (encoding 2-dehydro-3-deoxy-D-gluconate 5-dehydrogenase), COQ5 (encoding 2-methoxy-6-polyprenyl-1,4-benzoquinol methylase), *malT* (3′3′-cGAMP-specific phosphodiesterase 3), *rbsA* (encoding arabinose import ATP-binding protein AraG), and *gsiD* (encoding dipeptide transport system permease protein DppC) (Fig. 6C).

### Endophytic microbiome functional enrichment among banana cultivars

**Fig. 7:**
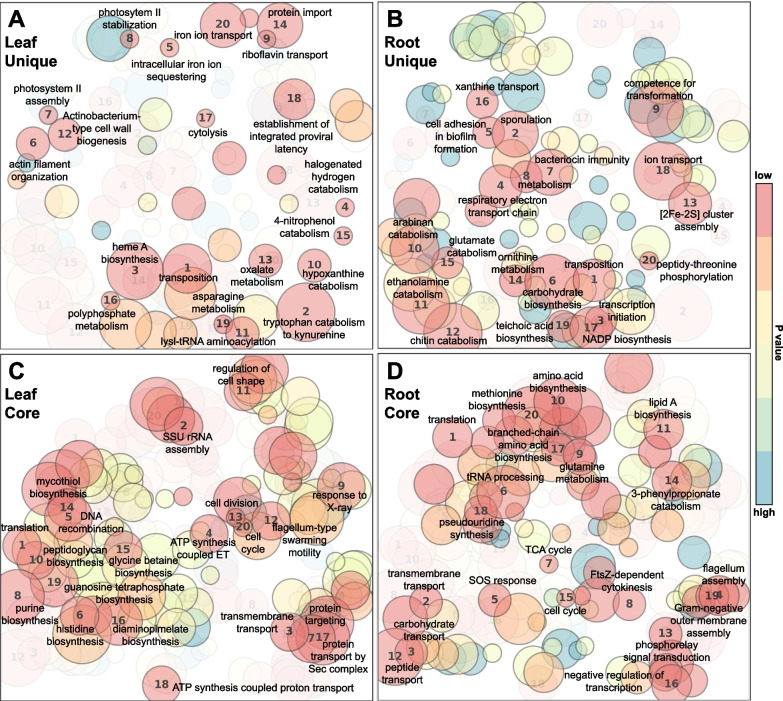
Patterns of significantly enriched gene ontology (GO) categories of the endophytic microbiome genes from combined *Musa* genotypes shown in Table 1 that were that were unique to leaf (**A**), or root (**B**), or that were universally shared among all samples for leaf (**C**) or root (**D**), depicted using ‘GO-figure!’ which projects similarity of biological process terms on semantic space axes x and y. Faded overlays show differences between clusters of categories between A-B and C-D. Distance between circles represents differences in GO term sematic space, as determined through multidimensional scaling analysis. Size of circle is proportional to the number of GO terms in that group. Numbers 1–20 indicate the top 20 most significant (lowest p-values) for GO terms based on topGO outputs shown in Additional file 1: Tables S13, S14, S15, Additional file 5: Table S16

To examine differences in potential functions across sampled endophytic microbiomes, gene ontology (GO) enrichment functional analyses were performed on root versus leaf for 6 *Musa* genotypes combined, comparing enriched functions in genes unique to metapangenomes of root (Additional file 1: Table S12) versus leaf (Additional file 1: Table S13). Overall, predicted functions were largely non-overlapping (Fig. 7A, B). In the first of these comparisons, examining gene sets unique to metapangenomes of leaf, which included only genes not present in the root in any sample, few unique GO terms were significantly enriched (Additional file 1: Table S13). In leaf metapangenomes, functions uniquely enriched included ferroxidase activity and ferric iron binding, and tryptophan catabolism and arylformamidase activity (associated with tryptophan metabolism) (Fig. 7A). There were more genes in root samples not present in leaf in any sample, thus there were more functions uniquely enriched (Additional file 1: Table S12). These included potential defense functions such as chitin binding and catabolism (potentially anti-fungal), bacterial defense response, bacteriocin immunity, and serine-type endopeptidase activity (potentially anti-nematocidal). There was also enriched cell-adhesion involved in biofilm formation and enriched genes for bacterial pilus (involved in bacteria-plant interaction), and various enriched activities associated with plant tissue colonization such as arabinan endo-1,5-alpha-L-arabinosidase activity and arabinan catabolism, xylan catabolism, cellulase activity, and plant membrane cholesterol and ethanolamine catabolism. There were also enriched functions for spore formation, sporulation, spore walls, and asexual sporulation (Fig. 7B).

For functions unique to core universally shared genes among all 6 samples for root (Additional file 1: Table S14) or core genes shared among all leaf samples (Additional file 1: Table S15), there were 192 distinct significantly enriched GO terms, with 46 of these enriched functions being identical between root and leaf (Additional file 5: Table S16). Overall, predicted functions for these core genes shared among samples overlapped somewhat (Fig. 7C, D). The significantly enriched GO terms that overlapped between core metagenomes of root and leaf were higher-level cellular “housekeeping” functions (Additional file 5: Table S16), whereas leaf and root had distinct patterns in GO enrichment. Notable GO terms enriched in core leaf metagenomes (Additional file 1: Table S15) included mycothiol biosynthesis (which is an antioxidant and antibacterial thiol in actinomycetes), 3 terms for viral and proviral integration, several terms for potential host-endophyte interaction or specialized function within plants (e.g., inositol phosphate dephosphorylation, which is associated with plant stress tolerance and adaptation, and biosynthesis of porphyrin compounds and diaminopimelate which are involved in heme and lysine synthesis, which are limiting in plant tissues), and other specialized functions such as cobalamin binding (often associated with N_2_ fixation) and glycine/betaine biosynthesis (associated with osmoprotection) (Fig. 7C). Among the 116 GO terms enriched in core root metagenomes (Additional file 1: Table S14), notable terms include functions related to xenobiotic transporter activity and xenobiotic detoxification, functions related to plant stress/defense such as polyamine transport and binding and putrescine catabolism, functions related to plant colonization such as rhamnose catabolism, chemotaxis, cobalamin biosynthesis, cellular response to H_2_O_2_ and oxidative stress, iron ion transport and homeostasis, nitrate assimilation, and virulence or pathogenic functions such as phenylacetate catabolism, enterobacterial common antigen biosynthesis, and 2 terms for response to radiation (Additional file 1: Table S14) (Fig. 7D).

Functional enrichment GO terms were distinctly different in comparisons of ‘wild’ diploids (samples BB, MB, MT, MS) and cultivated triploids (samples DC, FH) for both roots (Additional file 1: Tables S17 and S18) and leaves (Additional file 1: Tables S19 and S20). Notably, root endospheres of diploids were enriched for antimicrobial functions such as chitin catabolism and bacteriocin immunity, whereas leaf endospheres of diploids were enriched for antibiotic biosynthesis, biofilm formation, and xylan and glucan catabolism. In contrast, root and leaf endospheres of triploid cultivated banana had few functions enriched, although triploid roots did show enrichment for antibiotic biosynthesis. Similarly, GO term enrichment was distinctly different between AAA vs BB genotypes both above and below ground (Table 2). Root microbiomes of BB genotypes were enriched for penicillin binding, bacteriocin immunity, numerous defense functions, biofilm formation, pilus, sporulation, and chitin binding and catabolism, among others (Additional file 1: Table S21) whereas the AAA genotype was enriched for other processes, including cellulase, cellulose catabolism, nitrogen fixation, and toluene catabolism (Additional file 1: Table S22). Leaf microbiomes of BB genotypes were enriched for several activities including aromatic compound catabolism and response to toxic substance compared to AAA genotypes, which were enriched for sulfate assimilation, plasmid maintenance, and response to hypoxia among others (Additional file 1: Tables S23, S24). In similar comparisons between GO enrichment within each genotype among above and below ground tissues (BB: Additional file 1: Tables S25, S26; MB: Additional file 1: Tables S27, S28; MT: Additional file 1: Tables S29, S30; FH: Additional file 1: Tables S31, S32; DC: Additional file 1: Tables S33, S34; MS: Additional file 1: Tables S35, S36), each genotype displayed distinct functional enrichment.

**Table 2:** Key enriched gene ontology (GO) categories of the endophytic microbiome genes that were unique to metapangenomes from AAA-genotype (DC = *M. acuminata* Dwarf Cavendish) or BB-genotypes (BB = *M. balbisiana* ‘Thai Black’, MB = *M. balbisiana*) shown in Table 1, i.e., genes not shared among metapangenomes

AAA genotype	BB genotype
Leaves	Leaves
Sulfate assimilation	Metal ion binding
Phosphate ion transmembrane transport	Cell redox homeostasis
Plasmid maintenance	Aromatic compound catabolic process
Protein repair	Response to toxic substance
Response to iron ion	Endonuclease activity
Response to hypoxia	Iron-sulfur cluster binding
Hydrogen sulfide biosynthetic process	

Roots	Roots
Negative regulation of growth	Penicillin binding
Cellulase activity	Bacteriocin immunity
Cellulose catabolic process	Defense response to bacterium
Positive regulation of growth	Ethanolamine catabolic process
Sulfur cluster binding	Cell adhesion involved in single-species biofilm formation
rRNA catabolic process	Phenethylamine:oxygen oxidoreductase (deaminating) activity
Diacylglycerol O-acyltransferase activity	Protein secretion by the type III secretion system
3 Iron, 4 3-oxoacyl-[acyl-carrier-protein] synthase activity	Protein-phosphocysteine-sugar phosphotransferase activity
Host cell membrane	Establishment of competence for transformation
Nitrogen fixation	Modification-dependent protein catabolic process
Extracellular space	Chitin binding, chitin catabolic process
Endoribonuclease activity	Serine-type peptidase activity, peptidase activity
Toluene catabolic process	Cell death, cytolysis
	Pilus
	Sporulation resulting in formation of a cellular spore
	Spore germination, asexual sporulation
	Ion transmembrane transport
	Threonine-type endopeptidase activity
	Starch binding
	Serine-type endopeptidase activity
	Proteasomal protein catabolic process
	Glucose transmembrane transporter activity
	Phosphoenolpyruvate-dependent sugar phosphotransferase system
	Protein-N(PI)-phosphohistidine-sugar phosphotransferase activity
	Aminoacetone:oxygen oxidoreductase (deaminating) activity

### Analysis of orthologous clusters with signatures of phylosymbiosis

To assess whether shared homologous microbial gene clusters showed signatures of phylosymbiosis with host banana plants, we assessed phylogenetically the 718,012 individual gene alignment clusters from roary. From these, 3791 clusters had predicted gene homologs that included all 6 banana genotypes (BB, MB, FH, MT, MS, and DC), of which 205 clusters (5.4%), comprising 13,367 sequences, had tree topologies similar to that of their *Musa* hosts (i.e., (BB, MB), MT) and (DC, FH)) (Additional file 6: Table S37). Just 20 (~ 0.5%) of these clusters could only be annotated as ‘hypothetical protein’.

**Fig. 8:**
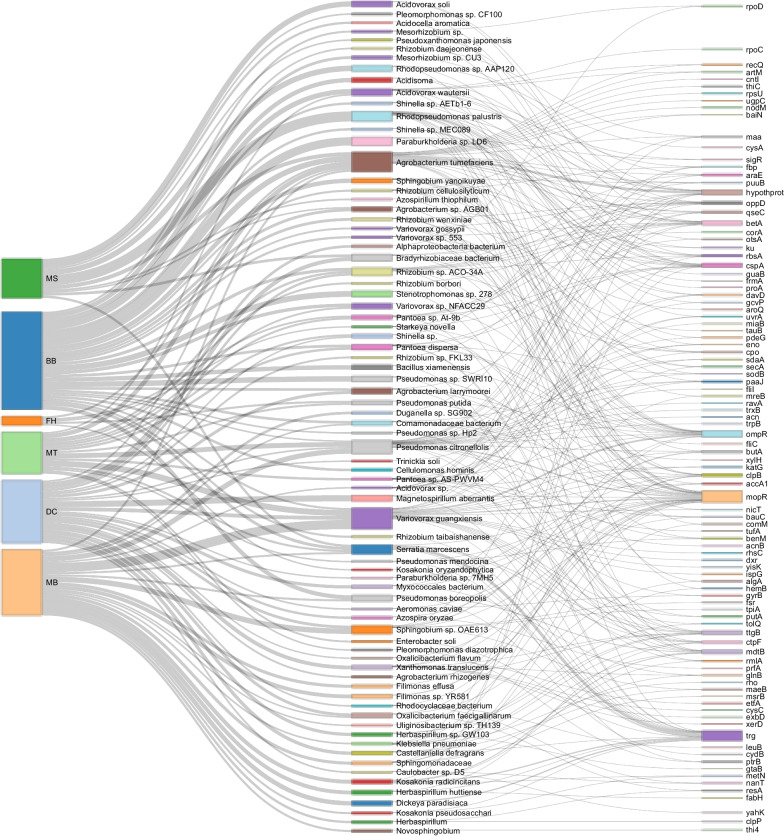
Associations between *Musa* genotypes (left) and abundant endophytic microbiome strains that displayed signatures of phylosymbiosis with their hosts (center) and genes forming the phylosymbiotic gene clusters (right). Size of block indicates relative abundance. *Musa* genotypes: BB = *M. balbisiana* ‘Thai Black’, DC = *M. acuminata* Dwarf Cavendish, FH = *M. acuminata* (FHIA-25), MB = *M. balbisiana*, MS = *M. sikkimensis*, MT = *M. textilis*. For display purposes, microbes without taxonomic annotation (i.e. microbes that could not be binned) and with fewer than 18 occurrences in the gene clusters were omitted, and genes corresponding to these microbes with fewer than 2 occurrences were omitted

This set of gene clusters revealed diverse taxa at the strain-level (Additional file 6: Table S37), including 2256 predicted strains, based on DIAMOND blast, and an additional 1944 sequences (46.3%) that had no taxonomic match to the nr databases (Additional file 1: Fig. S8). The most abundant taxa predicted to host these genes, from most to least abundant, were: *Variovorax guangxiensis*, *Agrobacterium tumefaciens*, *Pseudomonas citronellolis*, *Serratia marcescens*, *Rhodopseudomonas palustris*, *Rhizobium* sp. ACO-34A, *Paraburkholderia* sp. LD6, *Sphingobium* sp. OAE613, *Pseudomonas boreopolis*, *Bradyrhizobiaceae bacterium*, *Variovorax* sp. NFACC29, *Acidovorax wautersii*, *Stenotrophomonas* sp. 278, *Herbaspirillum huttiense*, and *Kosakonia radicincitans*. The most abundant genes, in order of abundance, amongst these clusters were *mopR* (encoding a phenol degradation regulator), *ompR* (encoding a DNA-binding dual transcriptional regulator involved in osmoregulation), hypothetical proteins of unknown function, *trg* (encoding a methyl-accepting chemotaxis protein III), *butA* (encoding diacetyl reductase [(S)-acetoin forming] which catalyzes 2,3-butanediol which can be found in some roots), *cspA* (encoding a cold shock protein), *betA* (encoding an oxygen-dependent choline dehydrogenase involved in the biosynthesis of the osmoprotectant glycine betaine), *ctpF* (encoding a cation-transporting ATPase F), *ttgB* (encoding a toluene efflux pump membrane transporter which also exports AMP and the antibiotics carbenicillin, nalidixic acid, chloramphenicol and tetracycline), *clpB* (encoding a chaperone protein that is part of the stress-induced system involved in the recovery of the cell from heat-induced damage), *rbsA* (encoding a ribose import ATP-binding protein), and *mdtB* (encoding a multidrug resistance protein that confers resistance against novobiocin and deoxycholate). Overall, *Musa* genotypes differed in the abundance and diversity of these microbes and the microbes differed in diversity of gene copies contributing to these gene clusters (Fig. 8). GO enrichment of these genes compared to the total set of genes in the samples showed significant enrichment for several functions, including SRP-dependent cotranslational protein targeting to membrane, removal of superoxide radicals, 3,4-dihydroxybenzoate catabolism, and bacteriocin transport (i.e., secretion of small antimicrobial peptides) (Additional file 1: Table S38).

## Discussion

This study analyzed cultivated and wild banana genotypes living in sympatry as a test of host-driven microbiome function. Our phylogenetic analysis of host plants matched previous studies [32, 56, 110] and showed that our sampled genotypes formed distinct clades spanning *Musa*. We predicted that if host genotype had little role in driving endosphere composition and function, endosphere metapangenomes would largely overlap. Instead, metapangenomes differed dramatically among hosts and between plant compartments, suggesting *Musa* hosts drive distinct microbiome-based disease protective functions.

A subset of gene clusters showed microbe-host phylogenomic similarity, which may reflect long-standing associations or heritable microbiomes similar to that found in other plants [106]. Among this gene set the dominant taxa, genes, and GO categories revealed novel findings worthy of more study. For example, the most abundant taxon was *Variovorax guangxiensis* (Burkholderiales) which was recently described from banana root and reported to produce the plant-growth promoting enzyme 1-aminocyclopropane-1-carboxylic acid deaminase (ACCd) [35]. Bacteria with ACCd are a major focus of research because they can buffer plant growth inhibition due to abiotic stress, by cleaving the stress response hormone ethylene [36, 83, 102]. However, curiously, the gene *acdS* for ACCd was not detected among the most abundant genes with phylosymbiosis in the present study, although the gene was detected and formed 11 distinct gene clusters across most samples. The next most abundant taxon was a strain of *Agrobacterium,* similar to *A. tumefaciens* or *Agrobacterium deltaense*, which is a widespread non-nodulating root growth-promoting symbiont thought to be an endophyte of interest [89]. *Pseudomonas citronellolis*, which is an ACCd possessing, alkane degrading, growth promoting bacterium [93] was also abundant. While there were many strains of *Pseudomonas* sp. in this data set, it is unclear if any of these might confer *Fusarium* wilt resistance such as that demonstrated for *Pseudomonas* sp. UPMP3 [33]. The next most-abundant species was *Serratia marcescens* which is a common endophyte with anti-pathogenic and anti-pest potential [49, 71]. Other abundant microbes with phylosymbiotic signals were *Rhodopseudomonas palustris, Rhizobium* sp. ACO-34A, *Pseudomonas boreopolis*, and *Stenotrophomonas* sp. 278, which are distinctive for banana soil disease protection, growth promotion via indole-3-acetic acid (IAA) biosynthesis, xylanase and antifungals production, and other potential *Fusarium* wilt protection [23, 28, 70, 82]. *Herbaspirillum huttiense* and *Kosakonia radicincitans* (syn. *Enterobacter radicincitans*, formerly *Pantoea agglomerans*) were also abundant, and may provide numerous plant-growth promoting and defense benefits [6, 11, 26]. Collectively, the most abundant genes with phylosymbiotic signal (e.g., *mopR, ompR, trg, butA, cspA, beta, ttgB, ctpF, clpB,* and *mdtB*) regulate phenol degradation, osmoregulation, chemotaxis, 2,3-butanediol catabolism, osmoprotectants, toluene and antibiotic efflux, and multidrug resistance, suggesting long-standing and specialized plant dependence on these genes. Moreover, GO enrichment for the full gene list suggested these phylosymbiotic banana microbial communities may function to benefit hosts through removal of superoxide radicals and secretion of small antimicrobial peptides (bacteriocin). Finally, almost two thousand sequences with phylosymbiotic signal in these clusters had no taxonomic match to databases whereas most genes had known function, revealing remarkable novelty in host-adapted endophytic taxa. This finding is consistent with previous work showing few endophytic bacteria are culturable [100].

Most gene clusters were distinct and not shared among different *Musa* genotypes, suggesting a strong influence of host genotype on endophytic community function. Other studies have shown host genotype was important for endosphere colonization in *Arabidopsis thaliana* and other plants [15, 34, 60, 109], while in algae over 70% of microbes were unique to host species [3]. Notably, these data are the first to uncover such patterns in banana. Furthermore, the majority of the over 500,000 predicted microbial gene clusters were annotated as ‘hypothetical protein’, suggesting a vast pool of novel microbiome genetic capacity that is largely unique to hosts and host tissues. This result is in contrast to past studies that were limited to microbes that were easily culturable [8], but is consistent with culture-free studies from other endophytic microbiomes, showing a dominance of genes of unknown function [20]. Others have shown genes of unknown function can be essential for endophytic colonization of plants [25]. In parallel with metagenomic diversity, our GO enrichment analyses showed differences among *Musa* genotypes. For example, we found enrichment for penicillin binding, bacteriocin immunity, numerous defense functions, biofilm formation, pilus, sporulation, and chitin binding and catabolism (in roots) and aromatic compound catabolism and response to toxic substances (in leaf) of BB genotypes compared to AAA genotypes, suggesting the wild banana genotype may retain a wealth of endophytic defenses. In contrast, the AAA genotype was enriched for cellulase, cellulose catabolism, nitrogen fixation, and toluene catabolism (in roots) and sulfate assimilation, plasmid maintenance, and response to hypoxia (in leaf) compared to BB genotypes, suggesting more antagonistic function or less diverse defenses in this cultivated plant. This result mirrors other studies showing more diverse rhizosphere microbiota associated with healthy banana plants [51] and richer microbial defense associated with resistant plants [64], but this is the first study exploring this in banana. Furthermore, GO analyses across *Musa* genotypes showed distinct patterns, revealing potentially diverse metapangenomic functional profiles among wild diploids *M. sikkimensis*, *M. textilis*, and *M. balbisiana*, and cultivated triploids AAB and AAA. Here, we also point out that although genotypes BB and MB were identical in the *ycf1* gene, these strains are still highly diverged [10, 65, 103], explaining their diverged microbiota. These findings should strengthen interest in these ‘crop wild relatives’ for novel microbiome-based bio-defenses.

Core functions across sampled *Musa* microbiomes included both familiar and novel enriched terms. Collectively, our microbiomes were enriched for familiar functions found in other plant microbiomes [1, 30, 91], including disease protection (antibiosis to phytopathogens and pests, beta-lactamase activity, chitinases, proteases, allelochemicals, siderophores, and ISR stimulation), plant growth promotion (phytohormone modulation such as via ACC deaminase and IAA biosynthesis, nutrient acquisition, nitrogen cycle, stress tolerance via glutathione, catalase, and peroxidase to detoxify reactive oxygen species, bioremediation of toxins, type III protein secretion, iron acquisition and storage), and colonization (chemotaxis, flagella, pili, polysaccharide adhesions, cellulases, pectinases, quorum sensing and biofilm formation, plant defense evasion, phosphotransferase system, ATP-binding cassette, catabolism of butanediol). But, beyond this, we found shared *Musa* core root metagenomes enriched for xenobiotic detoxification, polyamine transport and binding (presumably for defense), putrescine catabolism (presumably as a stress response), rhamnose catabolism (for plant colonization), cobalamin (vitamin B12) biosynthesis, phenylacetate catabolism (for defense), and response to radiation. These specific functional enrichments are novel for core plant root microbiomes. Similarly, core *Musa* leaf metagenomes were enriched for mycothiol synthesis (for defense in actinomycetes), proviral integration, inositol phosphate dephosphorylation (aiding plant stress tolerance), porphyrin and diaminopimelate synthesis (acting in heme and lysine synthesis which are limiting in plant tissues), and glycine/betaine synthesis (for osmoprotection). Together these metapangenomic analyses expand our growing knowledge of plant endospheres [1, 20, 30, 39] and greatly expand current targets [19, 44, 68, 76, 95, 98] for microbiome-based improvement of banana.

Beyond showing functional repertoire, our shotgun genomic data revealed over 24,000 distinct endophytic microbiome strains and novel taxonomic diversity patterns among genotypes and *Musa* tissues. However, a large portion of the reads (~ 20%) mapped to contigs that could not be binned taxonomically, whether the genes within them remained uncharacterized (as ‘hypothetical proteins’) or were functionally annotated, illuminating a wealth of uncharacterized diversity within banana plant tissues. A large proportion of taxa (~ 37%) occurred in only one tissue sampled i.e., ‘cloud’ taxa, with more than 2000 taxa unique to each of MB, BB, and DC genotypes, whereas just 0.76% of taxa were universally shared among all root and leaf samples, and these were mostly environmental and phytopathogenic species. This divergence of community among sympatric *Musa* specimens suggests the host drives the community composition. The same result has been reported previously in other plants [15, 34]. Cloud taxa varied among samples with MB and BB roots having the highest numbers of unique taxa, consistent with the pattern that crop wild relatives have divergent genomes and microbiomes [13, 57, 65, 68]. Although AAA genotypes were most different from one another in Bray–Curtis dissimilarity and there were more taxa unique to BB genotypes (~ 30%) than to AAA genotypes (~ 20%), suggesting BB plants have a greater capacity to recruit unique microbiota. Specifically, BB genotype plants had significantly more *Moraxellaceae*, *Luteimonas*, and *Solimonas*, while *M. sikkimensis* and *M. textilis* had more Rhizobiales, *Hoeflea*, and *Neorhizobium*, compared to FHIA-25 (AAB). *Corallococcus*, which may have remarkable antifungal potential [63] was enriched in AAB. These taxa have been analyzed in the context of disease protection: *Moraxellaceae* and *Luteimonas* (*Xanthomonadaceae*) in banana [68] and *Solimonas* in ISR [94], and the others are widely known as beneficial endophytes [1, 88, 108]. However, tissue was a stronger determinant of community composition: more taxa (16,360) were unique to roots while few taxa (690) were unique to tissues, with numerous taxa significantly more abundant in below ground tissues. This result is consistent with past studies in other plants [34, 109]. While many other common genera in these *Musa* samples *Kosakonia, Enterobacter, Klebsiella*, and *Citrobacter* (all *Enterobacteriaceae*), *Pantoea*, *Variovorax*, *Acidovorax*, *Paraburkholderia*, *Agrobacterium, Rhizobium*, *Pseudomonas*, *Strenotrophomonas*, and *Sphingobium*, mirroring findings by others [1, 44, 68, 99, 101], some genera were of special interest here. In particular, endophytes previously shown to have potential to control Panama disease by inhibiting *Fusarium oxysporum* f. sp. *cubense* activity [16, 19, 51, 68] varied across our *Musa* genotypes and specimens (e.g., *Chitinophaga*, *Chryseobacterium*, *Filimonas*, *Citrobacter*, *Stenotrophomonas*, *Enterobacter*, *Klebsiella*, *Kosakonia*, and some *Pseudomonas*). Although fungal reads were rarer in our data, one interesting and dominant fungal endophyte was *Podila* (syn. *Mortierella*) which can be an important endophyte [55, 74], and may be the host of the curious, protective endohyphal bacterium *Candidatus* Mycoavidus necroximicus [18] that we detected in some of our plants.

Not surprisingly, we found significant differences between root and leaf endospheres in terms of number of taxa, unique and undescribed taxa, and genes and functional enrichment, with roots being far richer than leaves for every *Musa* genotype. Given that the leaf endosphere largely derives from the roots, plant mediation of root colonization, e.g., through cracks in lateral root junctions that act as ‘doorways’ for colonization [22], may mechanistically explain our observed differences between both root and leaf microbiota. However, other studies [109] suggest adaptations in the microbes rather than the plants may control colonization. While finding richer taxa in roots would be expected, based on past studies, because roots are the source of microbiota and leaves present a more hostile environment [73, 97, 104], few studies have compared metapangenomes functionally. We found *Musa* root metapangenomes enriched for anti-fungal defense (e.g., chitin binding and catabolism), antibacterial defenses, and anti-nematocidal defenses (serine-type endopeptidase activity) and plant–microbe interactions (e.g., biofilm formation, pilus, and arabinan endo-1,5-alpha-L-arabinosidase activity, xylan catabolism, cellulase, and plant membrane catabolism), and spore forming functions, indicating a high degree of specialization for potentially beneficial roles. In contrast, unique metapangenomic features of leaf included tryptophan metabolism, ferroxidase, and ferric iron binding, which may reflect fewer benefits and more stressors affecting these tissues [73, 97, 104].

This study employed some alternative methods to produce novel results. First, our design focused on natural whole communities *in planta*, grown in sympatry in the same conditions in a small farm, thus presenting a natural experiment of the influence of host genotypes. By examining genes, strains, and predicted functions of these microbial communities, our outcomes presumably reflect potential inter-species synergies that have been demonstrated to be surprisingly dominant and enhanced by phylogenetic distances [48]. Second, our culture-free microbiome enrichment method, based on filtration and a Nycodenz density gradient, developed for soybean plants [43] enabled some special advantages. It facilitated analysis of a large volume of host tissue per sample, overcoming some of the unevenness of microbial microgeography within plants, while making the shotgun sequencing more cost effective by removing most of the plant material. Whereas our average plant DNA contamination was ~ 9.9%, without this enrichment, host DNA would have dominated the read output otherwise, limiting the depth of microbial reads. However, we found that host leaf DNA was highest in the Dwarf Cavendish leaf samples, suggesting possible lower bacteria-to-host abundance in the initial tissues. We also found a low volume yield of microbiome enrichment layer in Williams Hybrid (AAA) root and leaf samples, compared to all others, perhaps due to lower absolute microbiota abundance in these samples. We are unsure whether or how this might have affected the exceptionally low Illumina read yield for WH samples, but given the approximately equal ng DNA inputs into libraries and the standardized final library molarity normalizations, we speculate some possible intrinsic DNA compositional difference in the WH DNA libraries that may have affected the efficiency of cluster formation on the flow cells. We acknowledge one limitation of this enrichment method is that it has only been tested for bacteria. Although we found trace fungi, viruses, and archaea, we cannot be confident that those communities were represented proportionally and without taxonomic bias. Although a study of soil using Nycodenz density gradients suggested under-representation of Actinobacteria and Firmicutes [41], these microbes’ dense spore walls likely caused them to pellet rather than remain at the interface layer. We suggest that the absence of this finding by Ikeda et al. [43] may reflect much rarer mature spores relative to vegetative cells among endophytic cells, such that this bias is not present. Third, our shotgun sequencing and analysis methods including taxonomic abundances, gene repertoire metapangenomics, GO enrichment, phylosymbiosis analysis were efficient and powerful, enabling strain-level and gene-level resolution of differences, which is seldom possible with other methods [66]. One limitation of the functional analyses described here is that we were unable to assess the functional contribution of ‘hypothetical proteins’ or possible differences between annotated genes that grouped into separate homolog clusters. Addressing these challenges through new experimental and bioinformatic approaches will be critical to a deeper understanding of these complex microbiomes.

In conclusion, this study revealed key protective functions of the *Musa* endosphere are associated with host genotype, which may indicate a significant role for microbiome differences contributing to host resistance differences. Synergistic microbe-microbe-plant interactions could be important in observed disease resistance differences, including resistance to *Fusarium* wilt, between cultivars [7, 40, 67, 79]. Our data sets a baseline for future studies on these interactions, improving on historical studies that focused on isolated microbial strains or amplicon sequencing. We suggest future studies validate the functions predicted here, particularly from wild banana endophytic microbial consortia, with experiments *in planta*. We anticipate this will contribute to promising inocula to fortify defense mechanisms through in vivo bacterization [47] or engineering endophytes [58]. A very exciting outcome of our culture-free microbiome cell-enrichment is its future use in multi-omics, to overcome the barriers of quality and quantity of microbial mRNA and metabolites from full *in planta* endophytic communities.

## Supplementary Information

**Additional file 1**. Supplementary tables and figures.

**Additional file 2**. List of normalized read abundances of taxa detected with DIAMOND blastx in metagenomes assembled from 12 *Musa* samples showing unfiltered results, filtering for separate kingdoms, and showing taxon overlap results used in Venn diagrams.

**Additional file 3**. Pan-community analysis comparing the samples with the largest numbers of unique ‘cloud’ taxa, MBR, BBC, and DCR (which comprise BB and AAA genotypes), showing taxon overlap results used in Venn diagrams.

**Additional file 4**. List of all predicted genes across metapangenomes endophytic microbiomes from 12 sampled *Musa*, by genotype, including full gene clusters and consolidated genes with duplicate copies and unannotated genes removed, showing additional root/leaf and pairwise genotype Venn data.

**Additional file 5**. Enriched GO category results of shared and unshared root and leaf metapangenomes and core (shared) metapangenomes of root and leaf endophytic microbiomes from 6 *Musa* genotypes used in Venn diagrams.

**Additional file 6**. List of taxon matches and gene matches for which there was a signature of phylosymbiosis from for endophytic microbiomes of *Musa* samples, showing gene and taxon lists, counts, and complete DIAMOND blastx output.

## Data Availability

Sequence data and shotgun metagenomic assemblies may be accessed through NCBI accessions The datasets supporting the conclusions of this article are available in the NCBI repository, BioProject PRJNA837781, BioSamples SAMN28230877 to SAMN28230890 https://www.ncbi.nlm.nih.gov/search/all/?term=PRJNA837781.
