# Supplementary material for: Metapangenomics of wild and cultivated banana microbiome reveals a plethora of host-associated protective functions
Source: Environ Microbiome. 2023 Apr 21;18:36. doi: 10.1186/s40793-023-00493-x (PMC10120106; doi:10.1186/s40793-023-00493-x)
Supplement: Supplementary file 1 — Additional file 1. Supplementary tables and figures. [file 40793_2023_493_MOESM1_ESM.pdf]

Supplementary Tables and Figures

Supplementary Table 1: Comparison of number of sequenced reads that mapped to *Musa acuminata* and *Musa balbisiana* reference genomes for endophytic microbiomes from 14 sampled *Musa* cultivars and tissues. Sample names refer to the 14 *Musa* samples listed in **Table 1**.

	Musa acuminata			*Musa balbisiana*		
Sample	Merged	Paired	Total	Merged	Paired	Total
BBC	46 450 660	20 273 696	66 724 356	45 917 687	20 032 633	65 950 320
BBL	16 244 953	4 218 444	20 463 397	11 503 987	3 254 487	14 758 474
DCL	3 175 932	907 304	4 083 236	4 280 926	923 967	5 204 893
DCR	24 507 376	6 431 093	30 938 469	24 614 729	6 389 508	31 004 237
FHC	38 130 315	13 726 480	51 856 795	36 695 875	13 055 913	49 751 788
FHL	16 325 027	5 962 142	22 287 169	15 305 348	5 207 791	20 513 139
MBL	8 252 147	4 251 722	12 503 869	4 596 796	2 815 348	7 412 144
MBR	37 300 884	12 572 584	49 873 468	35 905 426	12 101 084	48 006 510
MSL	2 705 870	929 858	3 635 728	2 613 297	850 979	3 464 276
MSR	13 652 914	3 374 732	17 027 646	13 413 921	3 292 485	16 706 406
MTC	30 968 791	8 183 841	39 152 632	30 863 830	8 155 691	39 019 521
MTL	5 630 295	2 609 028	8 239 323	4 071 599	2 108 008	6 179 607
WHC	833 453	1 394 276	2 227 729	830 678	1 362 416	2 193 094
WHL	544 241	248 538	792 779	556 956	248 001	804 957
Sum	244 722 858	85 083 738	329 806 596	231 171 055	79 798 311	310 969 366

Supplementary Figure 1: Reads obtained from endophytic microbiomes from 14 sampled *Musa* cultivars and tissues, for (A) and (B) percentage of reads that mapped to 11 chromosomes of *Musa balbisiana* (DH-PKW), comparing samples grouped according to (A) leaf to (B) root tissues, and for (C) and (D) remaining reads (in millions) after quality-filtering and mapping to remove *Musa* reads for endophytic microbiomes for (C) leaf and (B) root (sequenced reads are represented by respective bar length whereas reads used for assembly are displayed as purple portions). Sample names and host genotypes are described on the x-axis.

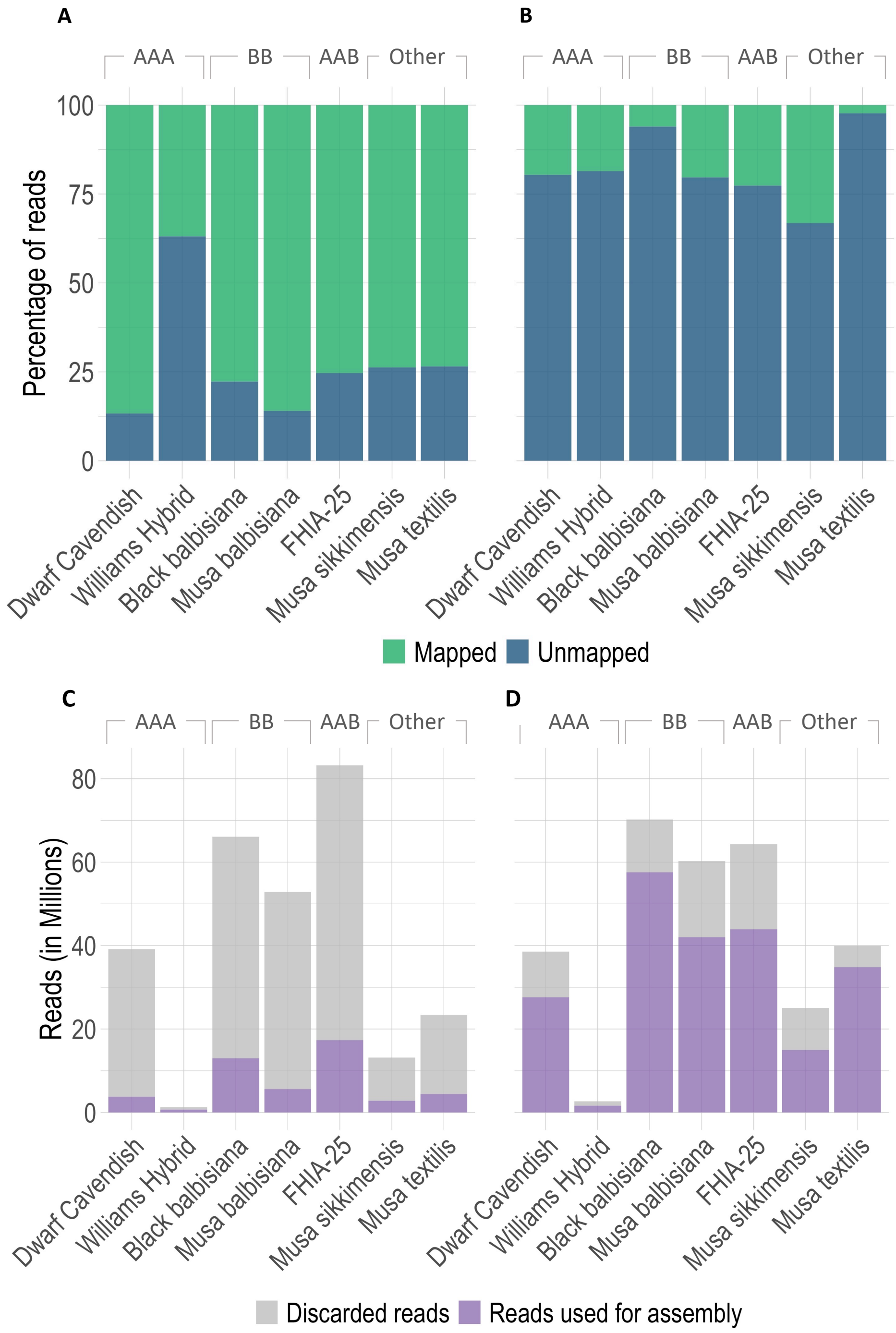
A
B
AAA
BB
AAB
Other
Percentage of reads
100
75
50
25
0
Dwarf Cavendish
Williams Hybrid
Black balbisiana
Musa balbisiana
FHIA-25
Musa sikkimensis
Musa textilis
Mapped
Unmapped
C
D
Reads (in Millions)
80
60
40
20
0
Discarded reads
Reads used for assembly

Supplementary Table 2: Assembly statistics for metagenomes generated from endophytic microbiomes for 14 sampled *Musa* cultivars and tissues, assessed using QUAST. Sample names refer to the 14 *Musa* samples listed in **Table 1**.

Sample	N50	Total length (Mb)	# Scaffolds	# Scaffolds >10kb	# Scaffolds >50kb	Largest scaffold (kbp)
BBC	1 829	495.81	493 020	3299	268	610.05
BBL	4 956	59.45	46 779	606	62	663.95
DCL	1 111	28.51	45 011	11	0	13.95
DCR	995	326.34	429 726	721	159	571.3
FHC	2 818	173.18	150 230	1 301	140	960.09
FHL	1 769	86.53	91 272	552	87	433.94
MBL	3 749	70.71	52 600	552	84	672.24
MBR	956	335.36	455 985	1 068	69	550.14
MSL	1 616	63.33	63 091	234	2	74.77
MSR	1 228	118.88	142 412	326	33	240.6
MTC	4 162	190.37	145 440	2 025	271	642.9
MTL	3 125	63.05	46 243	449	75	276.32
WHC	1 506	15.3	16 001	163	0	39.05
WHL	857	7.36	10 902	0	0	9.6

Supplementary Figure 2: Alpha rarefaction curves representing the observed number of OTUs versus the number of sampled sequences for endophytic microbiomes from 14 sampled Musa cultivars and tissues. Letters in boxes refer to the 14 Musa samples listed in **Table 1**.

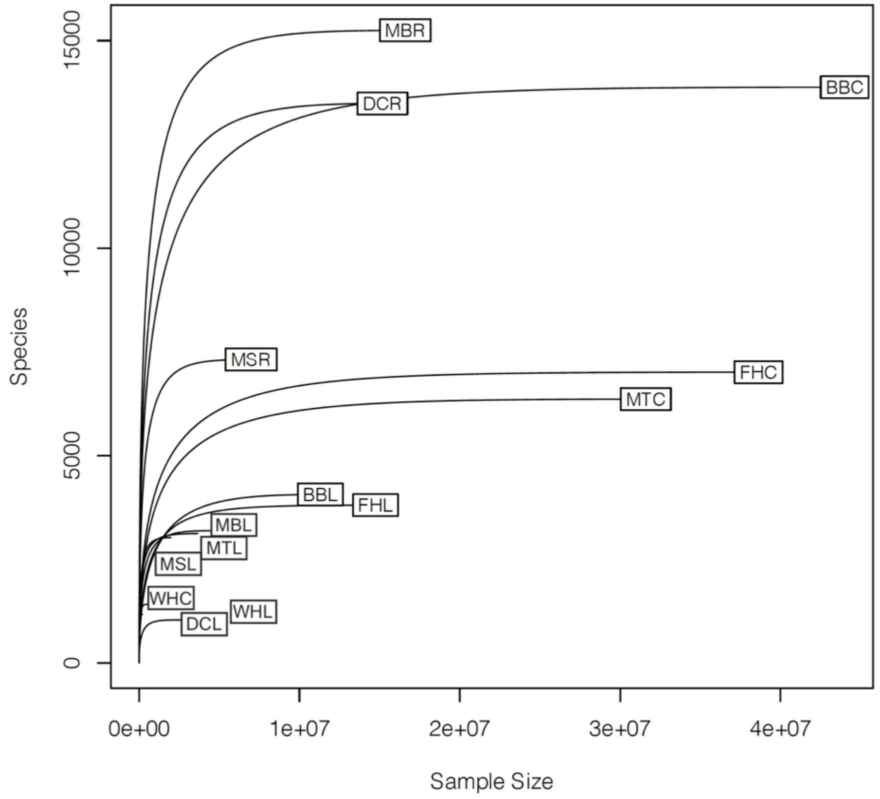

Supplementary Table 3: List of normalized read abundances of taxa detected with DIAMOND blastx in metagenomes assembled from 12 *Musa* samples (see additional excel file) showing unfiltered results, filtering for separate kingdoms, and showing taxon overlap results used in Venn diagrams.

Supplementary Figure 3: Number of shared and unshared taxa at lowest taxonomic levels (mostly species and strains) for endophytic microbiomes from 12 sampled *Musa* listed in **Table 1**, with (A) leaf and root compared individually, showing few universal 'core' taxa (black circle) occurring in every sample, compared to 'shell' taxa shared among two or more samples (grey circle) and unique unshared 'cloud' taxa (colored), and (B) communities for each *Musa* genotype combining tissues, leaf and root, showing each shared or unshared group via proportional area Venn drawing. Color legend for (A) and (B) indicates sampled genotypes BB = *M. balbisiana* 'Thai Black', DC = *M. acuminata* Dwarf Cavendish, FH = *M. acuminata* (FHIA-25), MB = *M. balbisiana*, MS = *M. sikkimensis*, MT = *M. textilis.*

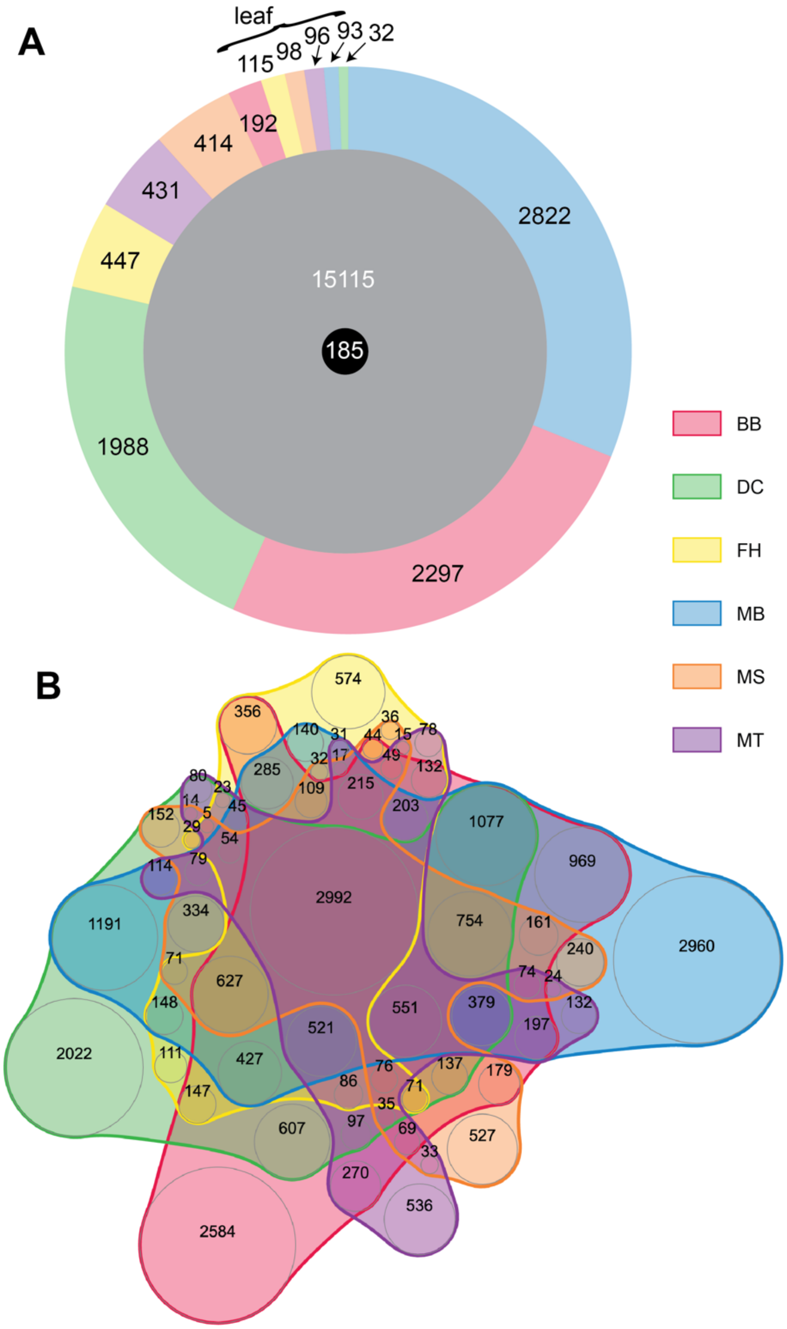

Supplementary Table 4: Pan-community analysis (see additional excel file) comparing the samples with the largest numbers of unique 'cloud' taxa, MBR, BBC, and DCR (which comprise BB and AAA genotypes), showing taxon overlap results used in Venn diagrams.

Supplementary Table 5: Taxonomic distribution of shotgun sequencing reads from endophytic microbiomes of 14 sampled *Musa* cultivars and tissues classified by proportion of reads mapping to scaffolds classified to superkingdom level by DIAMOND blast matches to the NCBI nr database. Sample names refer to the 14 *Musa* samples listed in **Table 1**.

Sample	Archaea	Bacteria	Fungi	Eukaryota (excluding fungi)	Viruses	Unclassified
BBC	762	37 771 951	570 279	1 403 106	8 429	4 257 053
BBL	14	5 909 428	4 848	2 023 489	19 484	3 340 617
DCL	6	40 593	997	586 878	12 364	1 945 959
DCR	23 250	12 418 597	59 511	505 411	2 909	2 167 819
FHC	68	20 915 106	1943 539	6 983 485	23 193	8 771 237
FHL	21	2 712 599	11 024	4 087 313	23 582	7 903 883
MBL	15	2 373 711	74 181	923 341	9 007	1 069 717
MBR	2 380	11 516 114	157 361	1 287 650	15 588	3 619 918
MSL	3	1 108 830	28 600	155 063	10 496	675 843
MSR	1 482	4 249 519	75 777	557 496	12 804	2 091 792
MTC	104	26 814 612	961 989	1 492 809	5 335	2 326 017
MTL	0	1 975 112	16 039	522 302	6 511	1 137 460
WHC	0	311 502	17 399	53 204	23	186 095
WHL	0	183 971	16	4 891	32	23 600
Sum	28 105	128 301 645	3 921 560	20 586 438	149 757	39 517 010

Supplementary Figure 4: Bacterial read counts from endophytic microbiomes of 14 sampled *Musa* cultivars and tissues for all samples (non-normalized) that were taxonomically assigned to level of (A) order or (B) genus by mapping to scaffolds classified based on DIAMOND blastn to the nr database, showing only taxa with more than 0.5% abundance.

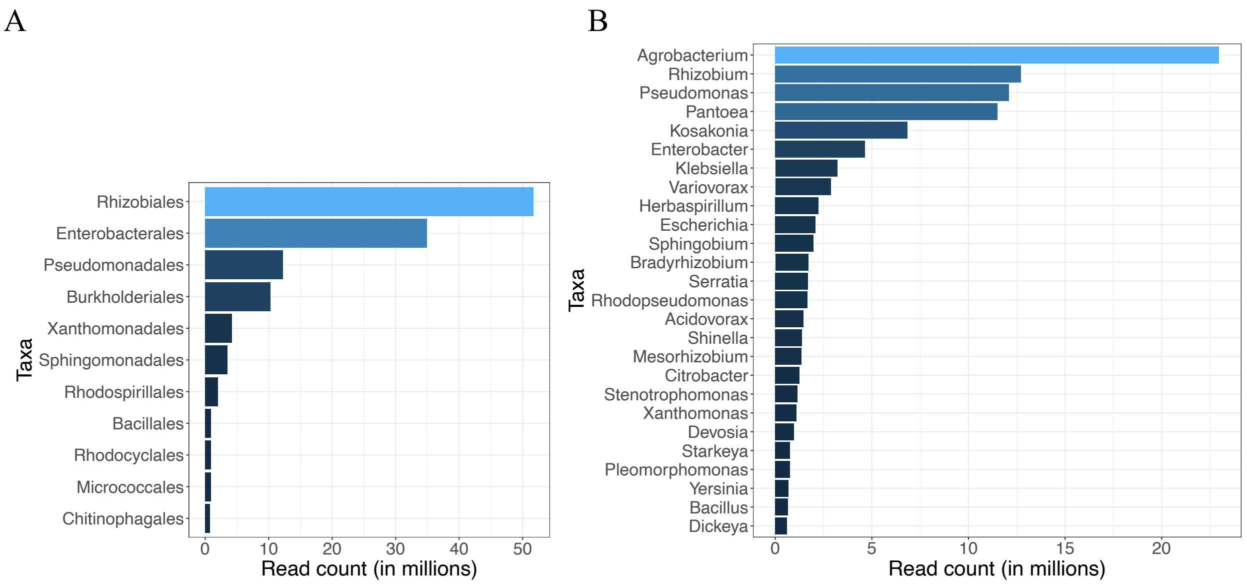

Supplementary Table 6: Bacterial taxa that were more abundant in below-ground (root) than above-ground (leaf) from endophytic microbiomes from sampled *Musa* cultivars and tissues for *Musa* samples listed in **Table 1** excluding DCL, WHC and WHL due to low sample sizes. P-values were from Welch's two sample t-tests analyzed *post hoc* following statistically significant ANOVA comparison between groups. Only taxa present in all samples were compared.

**Class**	**Order**	**Family**	**Genus**	**t-value**	**p-value**
Actinomycetia	Corynebacteriales	N/A	N/A	3.56	0.01
Actinomycetia	Corynebacteriales	Mycobacteriaceae	N/A	3.47	0.02
Alphaproteobacteria	Hyphomicrobiales	Parvibaculaceae	N/A	2.82	0.04
Alphaproteobacteria	Hyphomicrobiales	Methylobacteriaceae	N/A	2.33	0.05
Alphaproteobacteria	Hyphomicrobiales	Pleomorphomonadaceae	*Methylobrevis*	2.51	0.04
Alphaproteobacteria	Hyphomicrobiales	Beijerinckiaceae	N/A	2.65	0.04
Alphaproteobacteria	Hyphomicrobiales	Stappiaceae	*Stappia*	2.71	0.04
Alphaproteobacteria	Hyphomicrobiales	Kiastiaceae	*Kaistia*	3.52	0.02
Alphaproteobacteria	Hyphomicrobiales	Methylobacteriaceae	*Microvirga*	2.58	0.04
Alphaproteobacteria	Hyphomicrobiales	Bradyrhizobiaceae	*Bradyrhizobium*	2.94	0.03
Alphaproteobacteria	Hyphomicrobiales	Bradyrhizobiaceae	*Afipia*	2.67	0.04
Alphaproteobacteria	Hyphomonadales	N/A	N/A	2.43	0.04
Alphaproteobacteria	Hyphomonadales	Hyphomonadaceae	N/A	2.43	0.04
Alphaproteobacteria	Rhodospirillales	N/A	N/A	2.72	0.04
Alphaproteobacteria	Rhodospirillales	Azospirillaceae	*Nitrospirillum*	3.39	0.02
Alphaproteobacteria	Rhodospirillales	Acetobacteraceae	N/A	2.7	0.04
Alphaproteobacteria	Rhodospirillales	Acetobacteraceae	*Granulibacte*	3.21	0.02
Betaproteobacteria	Burkholderiales	Comamonadaceae	*Variovorax*	3.18	0.02
Betaproteobacteria	Burkholderiales	Comamonadaceae	N/A	3.07	0.03
Betaproteobacteria	Burkholderiales	Burkholderiaceae	*Ralstonia*	3.57	0.02
Betaproteobacteria	Burkholderiales	Burkholderiaceae	*Cupriavidus*	3.05	0.03
Gammaproteobacteria	Xanthomonadales	Rhodanobacteraceae	*Dyella*	3.14	0.03

Supplementary Table 7: Bacterial taxa that were differentially abundant among BB and AAB and other *Musa* genotypes from endophytic microbiomes from sampled *Musa* cultivars and tissues for Musa samples listed in **Table 1,** showing ANOVA results and Welch's t-tests from *post hoc* comparisons among taxa. Moraxellaceae, *Luteimonas*, and *Solimonas* were more abundant in *M. balbisiana* BB samples whereas Rhizobiales, *Hoeflea*, and *Neorhizobium* were more abundant in *M. sikkimensis* and *M. textilis* compared to FHIA-25 (AAB).

**Order**	**Family**	**Genus**	**ANOVA p-value**	**Pairwise t-test p-value**		
				**AAB-BB**	**AAB-Other**	**BB-Other**
Rhizobiales	N/A	N/A	0.25		0.005	
Hyphomicrobiales	Phyllobacteriaceae	*Hoeflea*	0.21		0.03	
Hyphomicrobiales	Rhizobiaceae	*Neorhizobium*	0.43		0.05	
Pseudomonadales	Moraxellaceae	N/A	0.002			
Myxococcales	Myxococcaceae	*Corallococcus*	0.56		0.01	
Nevskiales	Sinobacteraceae	*Solimonas*	0.04			
Xanthomoadales	Xanthomonadaceae	*Luteimonas*	0.005	0.01		0.02

Supplementary Figure 5: Normalized abundances between samples for specific genera of Bacteroidetes and Gammaproteobacteria from endophytic microbiomes from sampled *Musa* cultivars that were previously observed to potentially inhibit *Fusarium oxysporum* f. sp. *cubense* activity. Letters on the horizontal axis refer to the 14 *Musa* samples listed in **Table 1**. Samples DCL, WHC and WHL were excluded due to small sample size.

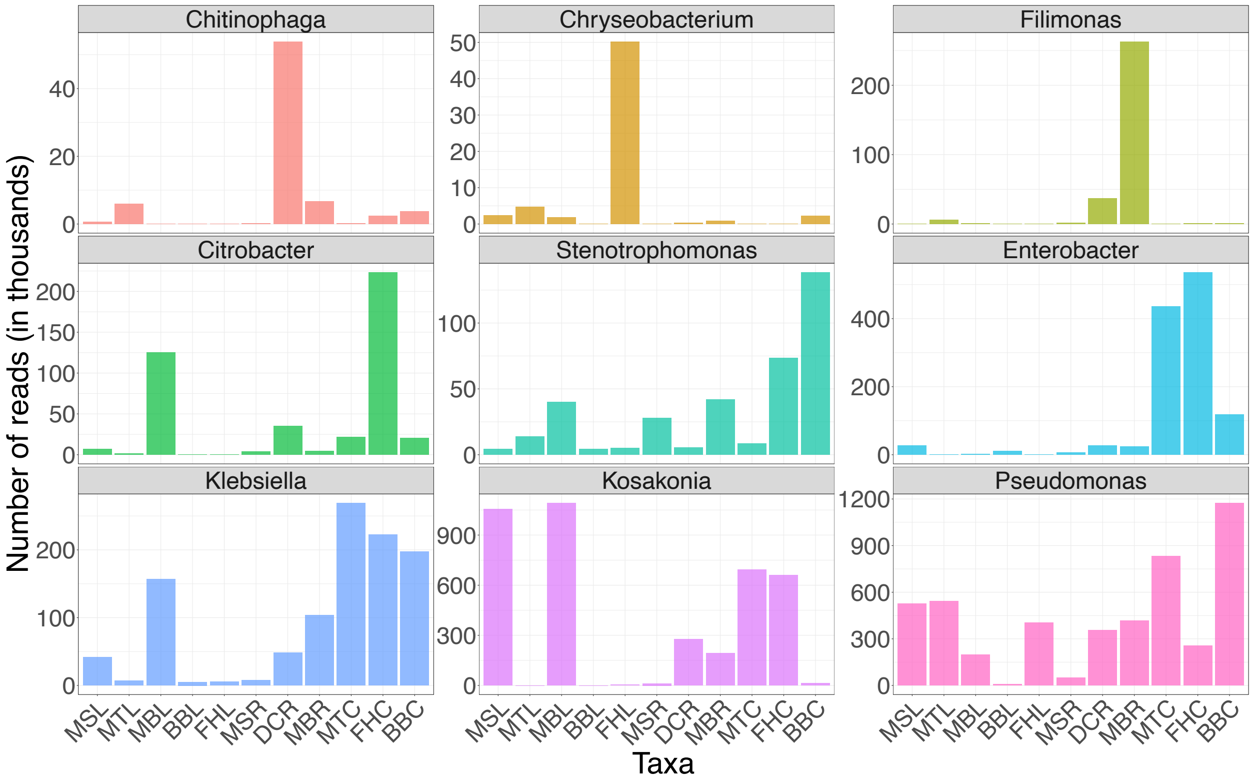

Supplementary Figure 6: Comparison of alpha diversity in endophytic microbiomes of 14 sampled *Musa* cultivars and tissues grouped according to (A, B) tissue location (leaves or roots) or (C, D) plant genotype (*M. acuminata* Dwarf Cavendish and Williams Hybrid = AAA, *M. acuminata* FHIA-25 = AAB, *M. balbisiana* and *M. balbisiana* 'Thai Black' = BB, *M. sikkimensis* and *M. textilis* = Others). Diversity was calculated by either the Chao1-index or ACE-index based on shotgun sequence reads mapped to taxonomically classified scaffolds.

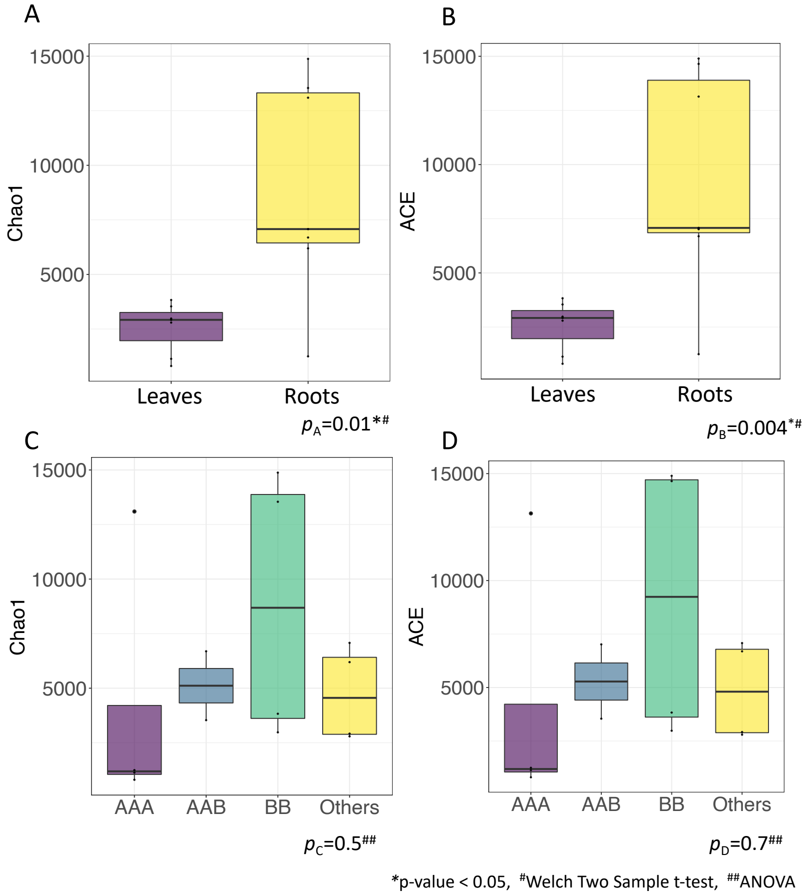

Supplementary Table 8: Alpha diversity indices for endophytic microbiomes of 14 sampled *Musa* cultivars and tissues for samples listed in **Table 1**. SVs are sequence variants generated in phyloseq based on shotgun sequence reads mapped to taxonomically classified scaffolds.

Sample	Observed SVs	Chao1	se.chao1	ACE	se.ACE	Shannon	Simpson	InvSimpson	Fisher
BBC	13543	14649.26	67.97	14704.98	59.79	5.4	0.98	50.24	1567.62
BBL	3829	3829	0.04	3829.52	30.89	2.31	0.77	4.28	387.93
DCL	804	804	0	804	4.63	0.25	0.05	1.06	69.75
DCR	13097	13139.73	8.25	13190.43	56.4	5.26	0.97	30.34	1509.49
FHC	6692	7016.95	31.93	7098.06	41.34	3.78	0.91	10.63	723.85
FHL	3536	3542.53	3.1	3550.54	29.46	1.79	0.45	1.81	360.17
MBL	2979	2979	0	2979	21.97	3.42	0.88	8.57	294.65
MBR	14877	14896.92	5.42	14921.87	59.55	5.28	0.94	16.47	1754.49
MSL	2794	2794	0	2794	9.68	3.48	0.84	6.39	269.99
MSR	7077	7077	0	7077	36.66	3.89	0.88	8.01	759.73
MTC	6195	6691.71	45.74	6691.48	40.32	4.29	0.97	28.99	652.07
MTL	2919	2919	0	2919	15.81	3.62	0.86	6.94	285.58
WHC	1247	1247	0	NA	NA	3.04	0.83	5.75	111.15
WHL	1133	1133	0	NA	NA	3.47	0.82	5.47	99.33

Supplementary Table 9: Ordination values based on Bray-Curtis dissimilarity among endophytic microbiomes from 14 sampled *Musa* cultivars and tissues listed in **Table 1** based on shotgun sequence reads mapped to taxonomically classified scaffolds.

Sample	Axis.1	Axis.2	Axis.3	Axis.4	Axis.5	Axis.6
BBC	0.372814	0.019434	0.000112	-0.131983	-0.140008	0.198154
BBL	-0.191006	0.056104	-0.028594	0.160933	-0.21589	0.11076
DCL	-0.401249	0.037994	0.060917	-0.234468	0.122027	0.057081
DCR	0.289755	0.051271	-0.24421	-0.051957	0.12364	0.00951
FHC	0.088003	-0.257474	0.179089	0.033634	0.008093	-0.099456
FHL	-0.301147	0.082167	0.061513	-0.132139	0.122938	0.082725
MBL	-0.089589	-0.255576	0.025574	0.257816	0.114559	0.071626
MBR	0.11496	-0.081341	-0.288359	-0.00685	0.080999	0.013922
MSL	-0.153009	0.025109	0.023791	0.082092	0.073175	-0.182239
MSR	-0.112102	-0.042066	-0.247677	-0.043689	-0.134868	-0.200375
MTC	0.409345	-0.134091	0.218502	-0.073084	0.052607	-0.02376
MTL	-0.15055	0.046771	0.005088	0.124905	-0.045134	0.167899
WHC	-0.078847	-0.025762	0.114971	-0.125905	-0.220134	-0.11467
WHL	0.202621	0.477459	0.119283	0.140694	0.057996	-0.091178
Sample	Axis.7	Axis.8	Axis.9	Axis.10	Axis.11	Axis.12
BBC	0.206746	-0.068856	-0.069413	-0.048225	-0.030924	0.016586
BBL	-0.197525	-0.043527	-0.144383	0.01662	-0.049931	-0.011659
DCL	-0.003421	0.018027	-0.098426	0.013255	0.026544	-0.0099
DCR	-0.163942	-0.026203	0.00297	-0.112797	0.083233	0.081647
FHC	0.001119	0.085112	-0.044053	0.073704	-0.056141	0.145312
FHL	0.000178	-0.006376	0.030049	0.032342	-0.012866	0.008576
MBL	0.10162	0.060832	-0.062779	-0.072351	0.106608	-0.054369
MBR	0.003398	0.162338	0.038725	0.050311	-0.145388	-0.067642
MSL	0.056137	-0.205117	0.024865	-0.124742	-0.118988	-0.007131
MSR	0.092794	-0.081807	-0.019753	0.126772	0.101182	-0.006289
MTC	-0.12902	-0.112499	0.037568	0.08368	0.024649	-0.098746
MTL	0.012681	-0.04876	0.246896	0.058003	0.021738	0.042455
WHC	-0.034852	0.16468	0.097771	-0.139675	0.02847	-0.033914
WHL	0.054086	0.102157	-0.040038	0.043102	0.021813	-0.004926

Supplementary Figure 7. Comparison of OTUs shared between endophytic microbiomes of 14 sampled *Musa* cultivars and tissues listed in **Table 1**.

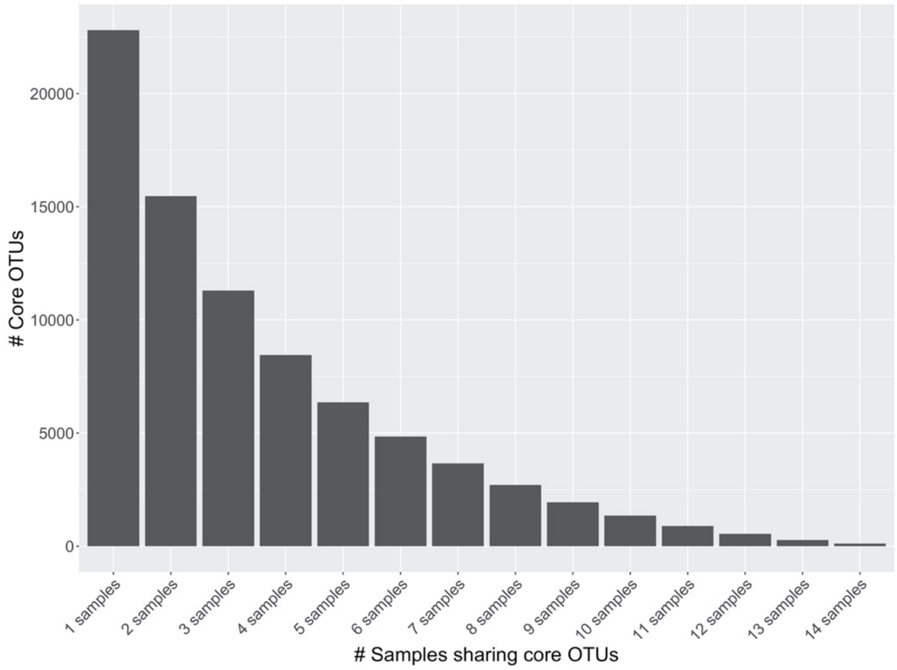

Supplementary Table 10: Number of predicted genes from endophytic microbiomes from 12 sampled *Musa* listed in **Table 1**, after normalizing scaffolds among samples, showing numbers of genes before and after removing redundant paralogs and genes of unknown function denoted 'hypothetical protein' by Prokka or 'group_#' by Roary.

Sample	Total Number of Genes Annotated	Number of Redundant Genes (i.e., Variants or Paralogs)	Number of Nonredundant Genes Including Genes of Unknown Function	Number of Genes of Unknown Function Denoted "group_#"	Number of Nonredundant Genes with Annotated Function
DCL	27 963	219	27 744	26 878	1 085
BBL	46 007	17 309	28 698	23 783	22 224
FHL	49 216	16 243	32 973	28 060	21 156
MSL	52 065	14 704	37 361	32 056	20 009
MTL	55 484	20 457	35 027	29 919	25 565
MBL	58 149	22 984	35 165	29 668	28 481
FHC	87 640	44 869	42 771	36 432	51 208
MTC	113 305	61 033	52 272	45 788	67 517
MSR	121 442	27 694	93 748	87 779	33 663
MBR	148 078	54 121	93 957	86 901	61 177
DCR	158 841	58 841	100 000	92 762	66 079
BBC	201 024	100 889	100 135	92 318	108 706

Supplementary Table 11: List of all predicted genes (see additional excel file) across metapangenomes endophytic microbiomes from 12 sampled *Musa*, by genotype, including full gene clusters and consolidated genes with duplicate copies and unannotated genes removed, showing additional root/leaf and pairwise genotype Venn data.

Supplementary Table 12: Significantly enriched gene ontology (GO) categories of the endophytic microbiome genes that were unique to the total root metapangenomes from 6 *Musa* genotypes shown in **Table 1** (BB = *M. balbisiana* 'Thai Black', DC = *M. acuminata* Dwarf Cavendish, FH = *M. acuminata* (FHIA-25), MB = *M. balbisiana*, MS = *M. sikkimensis*, MT = *M. textilis*), i.e., genes not shared with leaf metapangenomes.

GO_ID	Term	Annot-ated	Signif-icant	Expected	P-value
**Biological Processes:**					
GO:0006313	transposition, DNA-mediated	588	344	151.57	< 1e-30
GO:0015074	DNA integration	515	276	132.75	< 1e-30
GO:0030435	sporulation resulting in formation of a cellular spore	124	73	31.96	2.1e-16
GO:0006352	DNA-templated transcription, initiation	349	137	89.96	6.7e-09
GO:0022904	respiratory electron transport chain	158	61	40.73	1.2e-05
GO:0043709	cell adhesion involved in single-species biofilm formation	28	18	7.22	2.0e-05
GO:0009847	spore germination	9	8	2.32	0.00013
GO:0016051	carbohydrate biosynthetic process	234	39	60.32	0.00032
GO:0030153	bacteriocin immunity	11	8	2.84	0.00147
GO:0008152	metabolic process	5290	1402	1363.62	0.00157
GO:0032196	transposition	609	355	156.98	0.00280
GO:0030420	establishment of competence for transformation	25	12	6.44	0.00391
GO:0030436	asexual sporulation	10	7	2.58	0.00421
GO:0031222	arabinan catabolic process	8	6	2.06	0.00497
GO:0046336	ethanolamine catabolic process	6	5	1.55	0.00534
GO:0045493	xylan catabolic process	18	10	4.64	0.00679
GO:0006032	chitin catabolic process	9	6	2.32	0.01166
GO:0006707	cholesterol catabolic process	7	5	1.8	0.01472
GO:0044571	[2Fe-2S] cluster assembly	7	5	1.8	0.01472
GO:0006591	ornithine metabolic process	14	6	3.61	0.01750
GO:0071704	organic substance metabolic process	4651	1221	1198.9	0.03657
GO:0042742	defense response to bacterium	29	12	7.48	0.04808
**Cellular Components:**					
GO:0016021	integral component of membrane	2319	633	555.6	1.2e-11
GO:0005576	extracellular region	274	105	65.65	1.9e-08
GO:0009289	pilus	44	27	10.54	1.2e-07
GO:0031160	spore wall	11	9	2.64	0.00078
GO:0042601	endospore-forming forespore	5	4	1.2	0.01327
GO:0030076	light-harvesting complex	11	7	2.64	0.02257
**Metabolic Functions:**					
GO:0004803	transposase activity	587	344	147.67	< 1e-30
GO:0003677	DNA binding	1681	625	422.89	< 1e-30
GO:0003676	nucleic acid binding	2161	788	543.65	1.4e-24
GO:0016987	sigma factor activity	336	135	84.53	4.8e-10
GO:0009055	electron transfer activity	340	109	85.53	0.00046
GO:0008061	chitin binding	10	7	2.52	0.00362
GO:0046558	arabinan endo-1,5-alpha-L-arabinosidase activity	8	6	2.01	0.00436
GO:0031176	endo-1,4-beta-xylanase activity	9	6	2.26	0.01029
GO:0005509	calcium ion binding	44	18	11.07	0.01559

GO:0005355	glucose transmembrane transporter activity	6	5	1.51	0.01595
GO:0004534	5'-3' exoribonuclease activity	5	4	1.26	0.01597
GO:0016491	oxidoreductase activity	1634	385	411.07	0.02562
GO:0008810	cellulase activity	16	8	4.03	0.02801
GO:0004252	serine-type endopeptidase activity	53	20	13.33	0.02866

Supplementary Table 13: Significantly enriched gene ontology (GO) categories of the endophytic microbiome genes that were unique to the total leaf metapangenomes from 6 *Musa* genotypes shown in **Table 1** (BB = *M. balbisiana* 'Thai Black', DC = *M. acuminata* Dwarf Cavendish, FH = *M. acuminata* (FHIA-25), MB = *M. balbisiana*, MS = *M. sikkimensis*, MT = *M. textilis*), i.e., genes not shared with root metapangenomes.

GO_ID	Term	Annot-ated	Signif-icant	Expected	P-value
**Biological Processes:**					
GO:0006313	transposition, DNA-mediated	588	37	9.23	3.2e-14
GO:0015074	DNA integration	515	33	8.08	7.4e-13
GO:0019441	tryptophan catabolic process to kynurenine	10	2	0.16	0.010
GO:0032196	transposition	609	39	9.56	0.023
GO:0006310	DNA recombination	662	40	10.39	0.049
**Cellular Components:**					
GO:0016021	integral component of membrane	2319	37	27.38	0.0064
**Metabolic Functions:**					
GO:0004803	transposase activity	587	37	9.13	6.0e-14
GO:0003677	DNA binding	1681	49	26.14	2.4e-06
GO:0004322	ferroxidase activity	5	2	0.08	0.0023
GO:0004061	arylformamidase activity	7	2	0.11	0.0048
GO:0008199	ferric iron binding	15	2	0.23	0.0221
GO:0003676	nucleic acid binding	2161	60	33.61	0.0299
GO:0016746	acyltransferase activity	259	5	4.03	0.0333
GO:0004803	transposase activity	587	37	9.13	6.0e-14
GO:0003677	DNA binding	1681	49	26.14	2.4e-06
GO:0004322	ferroxidase activity	5	2	0.08	0.0023
GO:0004061	arylformamidase activity	7	2	0.11	0.0048
GO:0008199	ferric iron binding	15	2	0.23	0.0221
GO:0003676	nucleic acid binding	2161	60	33.61	0.0299
GO:0016746	acyltransferase activity	259	5	4.03	0.0333

Supplementary Table 14: Significantly enriched gene ontology (GO) categories of the endophytic microbiome genes that were universally shared among root samples from 6 *Musa* genotypes shown in **Table 1** (BB = *M. balbisiana* 'Thai Black', DC = *M. acuminata* Dwarf Cavendish, FH = *M. acuminata* (FHIA-25), MB = *M. balbisiana*, MS = *M. sikkimensis*, MT = *M. textilis*), i.e., core genes among root metapangenomes.

GO_ID	Term	Annot-ated	Signif-icant	Expected	P-value
**Biological Processes:**					
GO:0006412	translation	159	124	79.82	3.2e-09
GO:0055085	transmembrane transport	340	226	170.69	2.2e-08
GO:0008643	carbohydrate transport	131	80	65.77	7.5e-06
GO:0044780	bacterial-type flagellum assembly	22	20	11.04	3.2e-05
GO:0009432	SOS response	22	20	11.04	6.4e-05
GO:0008033	tRNA processing	52	45	26.11	0.00011
GO:0006099	tricarboxylic acid cycle	42	33	21.09	0.00014
GO:0043093	FtsZ-dependent cytokinesis	28	24	14.06	0.00028
GO:0006541	glutamine metabolic process	41	31	20.58	0.00029
GO:0008652	cellular amino acid biosynthetic process	211	157	105.93	0.00036
GO:0009245	lipid A biosynthetic process	18	16	9.04	0.00069
GO:0015833	peptide transport	45	35	22.59	0.00079
GO:0000160	phosphorelay signal transduction system	113	75	56.73	0.00095
GO:0019380	3-phenylpropionate catabolic process	10	10	5.02	0.00101
GO:0007049	cell cycle	105	79	52.71	0.00127
GO:0045892	negative regulation of transcription, DNA-templated	94	63	47.19	0.00163
GO:0009082	branched-chain amino acid biosynthetic process	28	23	14.06	0.00199
GO:0001522	pseudouridine synthesis	12	12	6.02	0.00201
GO:0043165	Gram-negative-bacterium-type cell outer membrane assembly	9	9	4.52	0.00201
GO:0009086	methionine biosynthetic process	31	24	15.56	0.00215
GO:0006189	'de novo' IMP biosynthetic process	16	14	8.03	0.00217
GO:0051301	cell division	109	81	54.72	0.00254
GO:0006749	glutathione metabolic process	33	27	16.57	0.00306
GO:0006096	glycolytic process	25	20	12.55	0.00345
GO:0006865	amino acid transport	105	72	52.71	0.00381
GO:0017004	cytochrome complex assembly	16	14	8.03	0.00383
GO:0009061	anaerobic respiration	21	16	10.54	0.00393
GO:0009435	NAD biosynthetic process	16	14	8.03	0.00400
GO:0032297	negative regulation of DNA-dependent DNA replication initiation	8	8	4.02	0.00402
GO:0045893	positive regulation of transcription, DNA-templated	71	49	35.64	0.00424
GO:0006260	DNA replication	95	68	47.69	0.00517
GO:0009246	enterobacterial common antigen biosynthetic process	11	10	5.52	0.00605
GO:0009228	thiamine biosynthetic process	14	12	7.03	0.00670
GO:0009229	thiamine diphosphate biosynthetic process	14	12	7.03	0.00670
GO:0006355	regulation of transcription, DNA-templated	394	241	197.8	0.00708
GO:0009116	nucleoside metabolic process	43	34	21.59	0.00974
GO:0000917	division septum assembly	22	18	11.04	0.00993

GO:0010124	phenylacetate catabolic process	19	15	9.54	0.00998
GO:0000105	histidine biosynthetic process	16	13	8.03	0.01102
GO:0031564	transcription antitermination	10	9	5.02	0.01106
GO:0071973	bacterial-type flagellum-dependent cell motility	47	34	23.6	0.01359
GO:0046777	protein autophosphorylation	24	18	12.05	0.01380
GO:0042128	nitrate assimilation	27	20	13.55	0.01506
GO:0009236	cobalamin biosynthetic process	48	32	24.1	0.01542
GO:0000270	peptidoglycan metabolic process	96	61	48.19	0.01569
GO:0006261	DNA-dependent DNA replication	37	29	18.57	0.01576
GO:0000028	ribosomal small subunit assembly	6	6	3.01	0.01597
GO:0015920	lipopolysaccharide transport	6	6	3.01	0.01597
GO:0000162	tryptophan biosynthetic process	6	6	3.01	0.01597
GO:0006744	ubiquinone biosynthetic process	19	15	9.54	0.01598
GO:0045454	cell redox homeostasis	23	17	11.55	0.01803
GO:0009306	protein secretion	75	41	37.65	0.01817
GO:0009447	putrescine catabolic process	9	8	4.52	0.02004
GO:0008360	regulation of cell shape	77	48	38.66	0.02094
GO:0009058	biosynthetic process	2225	1279	1117.01	0.02578
GO:0006974	cellular response to DNA damage stimulus	185	124	92.87	0.02698
GO:0006520	cellular amino acid metabolic process	427	287	214.36	0.02847
GO:0006351	transcription, DNA-templated	750	358	376.52	0.03150
GO:0006119	oxidative phosphorylation	27	21	13.55	0.03160
GO:0015846	polyamine transport	10	10	5.02	0.03172
GO:0008616	queuosine biosynthetic process	8	8	4.02	0.03177
GO:0006072	glycerol-3-phosphate metabolic process	10	9	5.02	0.03177
GO:0019634	organic phosphonate metabolic process	12	9	6.02	0.03182
GO:0035999	tetrahydrofolate interconversion	5	5	2.51	0.03184
GO:2000144	positive regulation of DNA-templated transcription, initiation	5	5	2.51	0.03184
GO:0018104	peptidoglycan-protein cross-linking	5	5	2.51	0.03184
GO:0006101	citrate metabolic process	5	5	2.51	0.03184
GO:0070301	cellular response to hydrogen peroxide	5	5	2.51	0.03184
GO:0036460	cellular response to cell envelope stress	5	5	2.51	0.03184
GO:0019301	rhamnose catabolic process	5	5	2.51	0.03184
GO:0090071	negative regulation of ribosome biogenesis	5	5	2.51	0.03184
GO:0006979	response to oxidative stress	70	50	35.14	0.03285
GO:0009097	isoleucine biosynthetic process	19	14	9.54	0.03287
GO:0016226	iron-sulfur cluster assembly	27	17	13.55	0.03297
GO:0010212	response to ionizing radiation	17	14	8.53	0.03345
GO:0006526	arginine biosynthetic process	17	13	8.53	0.03352
GO:0005978	glycogen biosynthetic process	12	10	6.02	0.03356
GO:0006826	iron ion transport	34	21	17.07	0.03590
GO:0006298	mismatch repair	8	7	4.02	0.03597
GO:0006212	uracil catabolic process	8	7	4.02	0.03597
GO:0009088	threonine biosynthetic process	8	7	4.02	0.03597
GO:0009252	peptidoglycan biosynthetic process	66	43	33.13	0.03751
GO:0055072	iron ion homeostasis	75	41	37.65	0.03846
GO:0031460	glycine betaine transport	16	12	8.03	0.03960
GO:1990961	xenobiotic detoxification by transmembrane export across the plasma membrane	21	15	10.54	0.04054
GO:0006935	chemotaxis	50	33	25.1	0.04181
GO:0009314	response to radiation	44	29	22.09	0.04704
**Cellular Components:**					

GO:0005829	cytosol	442	343	239.64	7.1e-21
GO:0005737	cytoplasm	1410	948	764.45	5.2e-15
GO:0005887	integral component of plasma membrane	266	182	144.22	2.7e-06
GO:0043190	ATP-binding cassette (ABC) transporter complex	71	57	38.49	3.6e-06
GO:0022625	cytosolic large ribosomal subunit	23	22	12.47	1.5e-05
GO:0032153	cell division site	22	20	11.93	0.00043
GO:0009425	bacterial-type flagellum basal body	20	18	10.84	0.00238
GO:0032993	protein-DNA complex	51	41	27.65	0.00393
GO:0045261	proton-transporting ATP synthase complex, catalytic core F(1)	7	7	3.8	0.01372
GO:0005840	ribosome	71	61	38.49	0.01401
GO:0022627	cytosolic small ribosomal subunit	17	14	9.22	0.01562
GO:0009326	formate dehydrogenase complex	6	6	3.25	0.02533
GO:0005886	plasma membrane	1756	1023	952.04	0.03032
GO:0030288	outer membrane-bounded periplasmic space	122	76	66.14	0.04179
**Metabolic Functions:**					
GO:0005524	ATP binding	801	508	399.2	2.9e-16
GO:0003735	structural constituent of ribosome	68	58	33.89	8.8e-10
GO:0019843	rRNA binding	58	51	28.91	2.0e-09
GO:0003700	DNA-binding transcription factor activity	602	289	300.02	2.3e-09
GO:0000049	tRNA binding	43	39	21.43	1.3e-08
GO:0042626	ATPase-coupled transmembrane transporter activity	181	129	90.21	4.9e-07
GO:0042802	identical protein binding	235	158	117.12	9.0e-07
GO:0022857	transmembrane transporter activity	908	586	452.53	1.0e-06
GO:0000287	magnesium ion binding	228	149	113.63	1.2e-06
GO:0000976	transcription cis-regulatory region binding	87	66	43.36	5.8e-06
GO:0008270	zinc ion binding	186	121	92.7	1.7e-05
GO:0003723	RNA binding	230	173	114.63	3.6e-05
GO:0015293	symporter activity	73	52	36.38	5.0e-05
GO:0008408	3'-5' exonuclease activity	22	19	10.96	0.00012
GO:0001216	DNA-binding transcription activator activity	37	29	18.44	0.00034
GO:0005525	GTP binding	79	54	39.37	0.00063
GO:0046872	metal ion binding	1762	977	878.14	0.00066
GO:0003924	GTPase activity	37	28	18.44	0.00118
GO:0050660	flavin adenine dinucleotide binding	201	113	100.17	0.00127
GO:0000166	nucleotide binding	1360	856	677.79	0.00156
GO:0015344	siderophore uptake transmembrane transporter activity	13	12	6.48	0.00164
GO:0038023	signaling receptor activity	50	25	24.92	0.00166
GO:0051287	NAD binding	100	65	49.84	0.00344
GO:0002161	aminoacyl-tRNA editing activity	12	11	5.98	0.00379
GO:0050661	NADP binding	76	51	37.88	0.00439
GO:0000156	phosphorelay response regulator activity	31	23	15.45	0.00500
GO:0043023	ribosomal large subunit binding	11	10	5.48	0.00566
GO:0030145	manganese ion binding	53	36	26.41	0.00579
GO:0043022	ribosome binding	14	12	6.98	0.00622
GO:0003681	bent DNA binding	7	7	3.49	0.00762
GO:0052591	sn-glycerol-3-phosphate:ubiquinone-8 oxidoreductase activity	7	7	3.49	0.00762
GO:0042803	protein homodimerization activity	42	29	20.93	0.00908
GO:0046933	proton-transporting ATP synthase activity, rotational mechanism	10	9	4.98	0.01042

GO:0003774	cytoskeletal motor activity	10	9	4.98	0.01042
GO:1904680	peptide transmembrane transporter activity	25	20	12.46	0.01077
GO:0009982	pseudouridine synthase activity	11	11	5.48	0.01524
GO:0004362	glutathione-disulfide reductase (NADPH) activity	6	6	2.99	0.01530
GO:0015220	choline transmembrane transporter activity	6	6	2.99	0.01530
GO:0008753	NADPH dehydrogenase (quinone) activity	6	6	2.99	0.01530
GO:0008137	NADH dehydrogenase (ubiquinone) activity	28	20	13.95	0.01696
GO:0001217	DNA-binding transcription repressor activity	12	10	5.98	0.01870
GO:0030955	potassium ion binding	9	8	4.49	0.01903
GO:0008863	formate dehydrogenase (NAD+) activity	9	8	4.49	0.01903
GO:0047834	D-threo-aldose 1-dehydrogenase activity	17	13	8.47	0.02365
GO:0003887	DNA-directed DNA polymerase activity	27	19	13.46	0.02493
GO:0003678	DNA helicase activity	24	20	11.96	0.02757
GO:0003684	damaged DNA binding	16	12	7.97	0.02780
GO:0043546	molybdopterin cofactor binding	29	20	14.45	0.02930
GO:0042910	xenobiotic transmembrane transporter activity	33	25	16.45	0.03036
GO:0008198	ferrous iron binding	24	17	11.96	0.03065
GO:0019808	polyamine binding	6	6	2.99	0.03069
GO:0008695	3-phenylpropionate dioxygenase activity	5	5	2.49	0.03071
GO:0003857	3-hydroxyacyl-CoA dehydrogenase activity	5	5	2.49	0.03071
GO:0019003	GDP binding	5	5	2.49	0.03071
GO:0043565	sequence-specific DNA binding	160	111	79.74	0.03176
GO:0015276	ligand-gated ion channel activity	11	9	5.48	0.03185
GO:0050136	NADH dehydrogenase (quinone) activity	36	27	17.94	0.03407
GO:0043531	ADP binding	8	7	3.99	0.03439
GO:0003697	single-stranded DNA binding	16	12	7.97	0.03720
GO:0016787	hydrolase activity	1513	790	754.04	0.04133

Supplementary Table 15: Significantly enriched gene ontology (GO) categories of the endophytic microbiome genes that were universally shared among leaf samples from 6 *Musa* genotypes shown in **Table 1** (BB = *M. balbisiana* 'Thai Black', DC = *M. acuminata* Dwarf Cavendish, FH = *M. acuminata* (FHIA-25), MB = *M. balbisiana*, MS = *M. sikkimensis*, MT = *M. textilis*), i.e., core genes among leaf metapangenomes.

GO_ID	Term	Annot-ated	Signif-icant	Expected	P-value
**Biological Processes:**					
GO:0006412	translation	146	35	16.34	2.5e-06
GO:0000028	ribosomal small subunit assembly	6	5	0.67	9.4e-05
GO:0055085	transmembrane transport	285	50	31.9	0.00035
GO:0042773	ATP synthesis coupled electron transport	9	5	1.01	0.00148
GO:0006310	DNA recombination	296	20	33.13	0.00177
GO:0000105	histidine biosynthetic process	15	6	1.68	0.00394
GO:0006605	protein targeting	12	7	1.34	0.00405
GO:0006164	purine nucleotide biosynthetic process	42	14	4.7	0.00494
GO:0009252	peptidoglycan biosynthetic process	53	13	5.93	0.00537
GO:0008360	regulation of cell shape	61	14	6.83	0.00629
GO:0071978	bacterial-type flagellum-dependent swarming motility	12	5	1.34	0.00698
GO:0071973	bacterial-type flagellum-dependent cell motility	40	12	4.48	0.00778
GO:0051301	cell division	98	23	10.97	0.00832
GO:0010125	mycothiol biosynthetic process	5	3	0.56	0.01173
GO:0043953	protein transport by the Tat complex	5	3	0.56	0.01173
GO:0019285	glycine betaine biosynthetic process from choline	9	4	1.01	0.01234
GO:0019877	diaminopimelate biosynthetic process	9	4	1.01	0.01234
GO:0043952	protein transport by the Sec complex	9	4	1.01	0.01234
GO:0006189	'de novo' IMP biosynthetic process	14	5	1.57	0.01460
GO:0007049	cell cycle	95	23	10.63	0.01585
GO:0009116	nucleoside metabolic process	41	9	4.59	0.01679
GO:0046855	inositol phosphate dephosphorylation	7	4	0.78	0.02143
GO:0046718	viral entry into host cell	6	3	0.67	0.02152
GO:0075713	establishment of integrated proviral latency	6	3	0.67	0.02152
GO:0044826	viral genome integration into host DNA	6	3	0.67	0.02152
GO:0043093	FtsZ-dependent cytokinesis	27	7	3.02	0.02553
GO:0019646	aerobic electron transport chain	11	4	1.23	0.02695
GO:0006099	tricarboxylic acid cycle	40	9	4.48	0.02951
GO:0044780	bacterial-type flagellum assembly	22	6	2.46	0.02982
GO:0045892	negative regulation of transcription, DNA-templated	81	15	9.07	0.03305
GO:0065002	intracellular protein transmembrane transport	9	4	1.01	0.03444
GO:0019740	nitrogen utilization	10	4	1.12	0.03446
GO:0009088	threonine biosynthetic process	7	3	0.78	0.03457
GO:0006268	DNA unwinding involved in DNA replication	7	3	0.78	0.03457
GO:0006614	SRP-dependent cotranslational protein targeting to membrane	5	3	0.56	0.03464
GO:0009432	SOS response	23	6	2.57	0.03666
GO:0006351	transcription, DNA-templated	558	62	62.46	0.04965
**Cellular Components:**					

GO:0009425	bacterial-type flagellum basal body	19	10	2.39	0.00016
GO:0022625	cytosolic large ribosomal subunit	23	9	2.89	0.00116
GO:0005887	integral component of plasma membrane	224	44	28.14	0.00284
GO:0005840	ribosome	68	25	8.54	0.00317
GO:0005737	cytoplasm	1189	191	149.38	0.00359
GO:0045261	proton-transporting ATP synthase complex, catalytic core F(1)	7	4	0.88	0.00630
GO:0022627	cytosolic small ribosomal subunit	16	6	2.01	0.01005
GO:0033281	TAT protein transport complex	5	3	0.63	0.01620
GO:0032993	protein-DNA complex	46	14	5.78	0.02405
GO:0009376	HslUV protease complex	6	3	0.75	0.02941
GO:0005618	cell wall	89	17	11.18	0.03749
GO:0005829	cytosol	394	73	49.5	0.03846
GO:1990077	primosome complex	7	3	0.88	0.04675
**Metabolic Functions:**					
GO:0003735	structural constituent of ribosome	64	24	6.59	6.2e-09
GO:0019843	rRNA binding	53	23	5.46	4.0e-08
GO:0005524	ATP binding	692	106	71.24	7.8e-06
GO:0046933	proton-transporting ATP synthase activity, rotational mechanism	10	7	1.03	1.1e-05
GO:0003924	GTPase activity	35	13	3.6	2.2e-05
GO:0005525	GTP binding	73	20	7.52	3.0e-05
GO:0003774	cytoskeletal motor activity	9	6	0.93	7.4e-05
GO:0003899	DNA-directed 5'-3' RNA polymerase activity	11	7	1.13	0.00017
GO:0000049	tRNA binding	41	11	4.22	0.00223
GO:0031419	cobalamin binding	16	6	1.65	0.00377
GO:0001217	DNA-binding transcription repressor activity	12	5	1.24	0.00487
GO:0043022	ribosome binding	13	5	1.34	0.00725
GO:0008137	NADH dehydrogenase (ubiquinone) activity	24	7	2.47	0.00859
GO:0071916	dipeptide transmembrane transporter activity	9	4	0.93	0.00918
GO:0004129	cytochrome-c oxidase activity	14	5	1.44	0.01033
GO:0001216	DNA-binding transcription activator activity	32	8	3.29	0.01356
GO:0008955	peptidoglycan glycosyltransferase activity	11	4	1.13	0.02035
GO:0003700	DNA-binding transcription factor activity	430	48	44.27	0.02544
GO:0003681	bent DNA binding	7	3	0.72	0.02769
GO:0000976	transcription cis-regulatory region binding	75	14	7.72	0.03461
GO:0003746	translation elongation factor activity	13	4	1.34	0.03736
GO:0042626	ATPase-coupled transmembrane transporter activity	162	31	16.68	0.04361
GO:0047834	D-threo-aldose 1-dehydrogenase activity	14	4	1.44	0.04817
GO:0003684	damaged DNA binding	14	4	1.44	0.04817

Supplementary Table 16: Enriched GO category results (see additional excel file) of shared and unshared root and leaf metapangenomes and core (shared) metapangenomes of root and leaf endophytic microbiomes from 6 *Musa* genotypes used in Venn diagrams.

Supplementary Table 17: Significantly enriched gene ontology (GO) categories of the endophytic microbiome genes that were unique to the root metapangenome from diploid 'wild' genotypes *Musa* (BB = *M. balbisiana* 'Thai Black', MB = *M. balbisiana*, MS = *M. sikkimensis*, MT = *M. textilis*) compared to cultivated triploid genotypes *Musa* (DC = *M. acuminata* Dwarf Cavendish, FH = *M. acuminata* (FHIA-25)) shown in **Table 1**, i.e., genes not shared with root triploid and triploid+diploid metapangenomes.

GO_ID	Term	Annot-ated	Signif-icant	Expected	P-value
**Biological Processes:**					
GO:0030435	sporulation resulting in formation of a cellular spore	119	35	9.6	5.50E-13
GO:0022904	respiratory electron transport chain	156	37	12.59	1.60E-09
GO:0006352	DNA-templated transcription, initiation	262	43	21.14	5.40E-06
GO:0030420	establishment of competence for transformation	25	9	2.02	2.10E-05
GO:0009847	spore germination	9	5	0.73	0.00032
GO:0042742	defense response to bacterium	27	8	2.18	0.00095
GO:0015979	photosynthesis	38	14	3.07	0.00338
GO:0006032	chitin catabolic process	9	4	0.73	0.0038
GO:0030436	asexual sporulation	10	4	0.81	0.00593
GO:0019835	cytolysis	30	7	2.42	0.00844
GO:0019253	reductive pentose-phosphate cycle	12	4	0.97	0.01227
GO:0036070	light-independent bacteriochlorophyll biosynthetic process	7	3	0.56	0.01428
GO:0019941	modification-dependent protein catabolic process	7	3	0.56	0.01428
GO:0018298	protein-chromophore linkage	13	4	1.05	0.01662
GO:0030153	bacteriocin immunity	8	3	0.65	0.0215
GO:0019684	photosynthesis, light reaction	8	3	0.65	0.0215
GO:0010498	proteasomal protein catabolic process	9	3	0.73	0.03035
GO:0015995	chlorophyll biosynthetic process	16	7	1.29	0.03433
GO:0019685	photosynthesis, dark reaction	16	6	1.29	0.03448
**Cellular Components:**					
GO:0016021	integral component of membrane	1944	204	166.98	4.40E-07
GO:0030076	light-harvesting complex	11	9	0.94	8.90E-06
GO:0005576	extracellular region	235	39	20.19	2.70E-05
GO:0019866	organelle inner membrane	7	3	0.6	0.01693
GO:0005886	plasma membrane	1690	156	145.16	0.01884
**Metabolic Functions:**					
GO:0009055	electron transfer activity	309	56	23.96	7.90E-09
GO:0016987	sigma factor activity	251	42	19.46	1.20E-06
GO:0003677	DNA binding	956	91	74.13	0.00032
GO:0008233	peptidase activity	246	36	19.08	0.0005
GO:0004252	serine-type endopeptidase activity	46	10	3.57	0.00229
GO:0016984	ribulose-bisphosphate carboxylase activity	9	3	0.7	0.02735
GO:0008061	chitin binding	10	3	0.78	0.03686
GO:0042834	peptidoglycan binding	10	3	0.78	0.03686
GO:0004803	transposase activity	11	3	0.85	0.04784

Supplementary Table 18: Significantly enriched gene ontology (GO) categories of the endophytic microbiome genes that were unique to the root metapangenome from cultivated triploid genotypes *Musa* (DC = *M. acuminata* Dwarf Cavendish, FH = *M. acuminata* (FHIA-25)) compared to diploid 'wild' genotypes *Musa* (BB = *M. balbisiana* 'Thai Black', MB = *M. balbisiana*, MS = *M. sikkimensis*, MT = *M. textilis*) shown in **Table 1**, i.e., genes not shared with root diploid and triploid+diploid metapangenomes.

GO_ID	Term	Annot-ated	Signif-icant	Expected	P-value
**Biological Processes:**					
GO:0009399	nitrogen fixation	34	5	0.91	0.0019
GO:0017000	antibiotic biosynthetic process	68	7	1.82	0.0021
GO:0042203	toluene catabolic process	13	3	0.35	0.0044
GO:0010207	photosystem II assembly	5	2	0.13	0.0067
GO:0042549	photosystem II stabilization	5	2	0.13	0.0067
GO:0006352	DNA-templated transcription, initiation	262	14	7	0.0098
GO:0006265	DNA topological change	7	2	0.19	0.0136
GO:0032259	methylation	103	7	2.75	0.0197
**Cellular Components:**					
GO:0005576	extracellular region	235	15	6.14	0.0034
GO:0031676	plasma membrane-derived thylakoid membrane	5	2	0.13	0.0064
GO:0009654	photosystem II oxygen evolving complex	5	2	0.13	0.0064
GO:0033644	host cell membrane	12	2	0.31	0.0376
**Metabolic Functions:**					
GO:0051538	3 iron, 4 sulfur cluster binding	14	3	0.39	0.00616
GO:0010242	oxygen evolving activity	5	2	0.14	0.00729
GO:0016987	sigma factor activity	251	14	6.99	0.01001
GO:0003690	double-stranded DNA binding	108	5	3.01	0.01717
GO:0004315	3-oxoacyl-[acyl-carrier-protein] synthase activity	24	3	0.67	0.02794
GO:0031177	phosphopantetheine binding	26	3	0.72	0.03447
GO:0004144	diacylglycerol O-acyltransferase activity	11	2	0.31	0.03592
GO:0051537	2 iron, 2 sulfur cluster binding	113	7	3.14	0.03768
GO:0005507	copper ion binding	47	4	1.31	0.04091

Supplementary Table 19: Significantly enriched gene ontology (GO) categories of the endophytic microbiome genes that were unique to the leaf metapangenome from diploid 'wild' genotypes *Musa* (BB = *M. balbisiana* 'Thai Black', MB = *M. balbisiana*, MS = *M. sikkimensis*, MT = *M. textilis*) compared to cultivated triploid genotypes *Musa* (DC = *M. acuminata* Dwarf Cavendish, FH = *M. acuminata* (FHIA-25)) shown in **Table 1**, i.e., genes not shared with leaf triploid and triploid+diploid metapangenomes.

GO_ID	Term	Annot-ated	Signif-icant	Expected	P-value
**Biological Processes:**					
GO:0009401	phosphoenolpyruvate-dependent sugar phosphotransferase system	51	32	13.03	1.90E-08
GO:0022904	respiratory electron transport chain	95	44	24.27	1.30E-07
GO:0017000	antibiotic biosynthetic process	46	25	11.75	0.00014
GO:0043709	cell adhesion involved in single-species biofilm formation	10	7	2.55	0.00396
GO:0030254	protein secretion by the type III secretion system	8	6	2.04	0.00472
GO:0045490	pectin catabolic process	15	9	3.83	0.00484
GO:0000272	polysaccharide catabolic process	57	34	14.56	0.00571
GO:0015979	photosynthesis	30	16	7.66	0.0063
GO:0019290	siderophore biosynthetic process	19	12	4.85	0.00838
GO:0009297	pilus assembly	15	9	3.83	0.01165
GO:0009399	nitrogen fixation	28	13	7.15	0.01326
GO:0045493	xylan catabolic process	7	5	1.79	0.01412
GO:1902201	negative regulation of bacterial-type flagellum-dependent cell motility	7	5	1.79	0.01412
GO:0071805	potassium ion transmembrane transport	6	5	1.53	0.01658
GO:0030494	bacteriochlorophyll biosynthetic process	10	7	2.55	0.01679
GO:0030980	alpha-glucan catabolic process	5	4	1.28	0.01689
GO:0006352	DNA-templated transcription, initiation	160	51	40.87	0.02416
GO:0006071	glycerol metabolic process	26	10	6.64	0.02712
GO:0019605	butyrate metabolic process	8	5	2.04	0.02978
GO:0000746	conjugation	12	7	3.07	0.03766
GO:0015977	carbon fixation	14	8	3.58	0.0402
GO:1901848	nicotinate catabolic process	6	4	1.53	0.04044
**Cellular Components:**					
GO:0016021	integral component of membrane	1483	456	389.52	5.40E-10
GO:0005576	extracellular region	147	64	38.61	2.20E-06
GO:0005886	plasma membrane	1346	386	353.54	0.00024
GO:0015627	type II protein secretion system complex	13	7	3.41	0.03131
**Metabolic Functions:**					
GO:0008982	protein-N(PI)-phosphohistidine-sugar phosphotransferase activity	26	18	6.78	2.10E-06
GO:0009055	electron transfer activity	211	79	54.99	7.20E-05
GO:0016984	ribulose-bisphosphate carboxylase activity	8	7	2.08	0.0005
GO:0051536	iron-sulfur cluster binding	251	99	65.41	0.00057
GO:0015473	fimbrial usher porin activity	9	7	2.35	0.00174
GO:0051539	4 iron, 4 sulfur cluster binding	131	49	34.14	0.00254
GO:0004556	alpha-amylase activity	10	7	2.61	0.00449
GO:0016163	nitrogenase activity	6	5	1.56	0.00562
GO:0030570	pectate lyase activity	6	5	1.56	0.00562

GO:0016298	lipase activity	18	7	4.69	0.00564
GO:0046872	metal ion binding	1306	369	340.36	0.00822
GO:0008234	cysteine-type peptidase activity	11	8	2.87	0.01226
GO:0031177	phosphopantetheine binding	17	9	4.43	0.01621
GO:0004190	aspartic-type endopeptidase activity	12	7	3.13	0.01788
GO:0004494	methylmalonyl-CoA mutase activity	5	4	1.3	0.01821
GO:2001070	starch binding	5	4	1.3	0.01821
GO:0051537	2 iron, 2 sulfur cluster binding	92	33	23.98	0.0231
GO:0016987	sigma factor activity	150	50	39.09	0.02692
GO:0005506	iron ion binding	117	34	30.49	0.02764
GO:0005509	calcium ion binding	24	11	6.25	0.02849
GO:0030976	thiamine pyrophosphate binding	30	13	7.82	0.02969
GO:0004497	monooxygenase activity	98	35	25.54	0.03785
GO:0008047	enzyme activator activity	8	6	2.08	0.04323
GO:0008564	protein-exporting ATPase activity	6	4	1.56	0.04339
GO:0103025	alpha-amylase activity (releasing maltohexaose)	6	4	1.56	0.04339
GO:0042602	riboflavin reductase (NADPH) activity	6	4	1.56	0.04339

Supplementary Table 20: Significantly enriched gene ontology (GO) categories of the endophytic microbiome genes that were unique to the leaf metapangenome from cultivated triploid genotypes *Musa* (DC = *M. acuminata* Dwarf Cavendish, FH = *M. acuminata* (FHIA-25)) compared to diploid 'wild' genotypes *Musa* (BB = *M. balbisiana* 'Thai Black', MB = *M. balbisiana*, MS = *M. sikkimensis*, MT = *M. textilis*) shown in **Table 1**, i.e., genes not shared with leaf diploid and triploid+diploid metapangenomes.

GO_ID	Term	Annot-ated	Signif-icant	Expected	P-value
**Biological Processes:**					
GO:0006048	UDP-N-acetylglucosamine biosynthetic process	5	2	0.09	0.0032
GO:0010207	photosystem II assembly	6	2	0.11	0.0048
GO:0042549	photosystem II stabilization	6	2	0.11	0.0048
GO:0030163	protein catabolic process	24	3	0.44	0.0193
**Cellular Components:**					
GO:0031676	plasma membrane-derived thylakoid membrane	6	2	0.08	0.0026
GO:0009654	photosystem II oxygen evolving complex	6	2	0.08	0.0026
GO:0005615	extracellular space	13	2	0.18	0.0126
**Metabolic Functions:**					
GO:0052874	FMN reductase (NADH) activity	5	2	0.09	0.00314
GO:0010242	oxygen evolving activity	6	2	0.11	0.00465
GO:0051213	dioxygenase activity	74	5	1.34	0.00979
GO:0008199	ferric iron binding	14	2	0.25	0.02567

Supplementary Table 21: Significantly enriched gene ontology (GO) categories of the endophytic microbiome genes that were unique to the root metapangenome from BB-genotype *Musa* (BB = *M. balbisiana* 'Thai Black', MB = *M. balbisiana*) compared to AAA-genotype *Musa* (DC = *M. acuminata* Dwarf Cavendish) shown in **Table 1**, i.e., genes not shared with root AAA and AAA+BB metapangenomes.

GO_ID	Term	Annot-ated	Signif-icant	Expected	P-value
**Biological Processes:**					
GO:0022904	respiratory electron transport chain	152	52	23.55	3.5e-10
GO:0030435	sporulation resulting in formation of a cellular spore	119	41	18.44	5.0e-08
GO:0043709	cell adhesion involved in single-species biofilm formation	26	15	4.03	9.1e-07
GO:0006352	DNA-templated transcription, initiation	253	67	39.2	2.8e-06
GO:0009401	phosphoenolpyruvate-dependent sugar phosphotransferase system	64	21	9.91	0.00041
GO:0030420	establishment of competence for transformation	25	10	3.87	0.00210
GO:0006032	chitin catabolic process	8	5	1.24	0.00328
GO:0019835	cytolysis	28	10	4.34	0.00328
GO:0030254	protein secretion by the type III secretion system	12	6	1.86	0.00542
GO:0009847	spore germination	9	5	1.39	0.00645
GO:0034220	ion transmembrane transport	91	14	14.1	0.00658
GO:0046336	ethanolamine catabolic process	6	4	0.93	0.00660
GO:0042742	defense response to bacterium	24	9	3.72	0.00716
GO:0030436	asexual sporulation	10	5	1.55	0.01126
GO:0019941	modification-dependent protein catabolic process	7	4	1.08	0.01353
GO:0008219	cell death	15	3	2.32	0.02404
GO:0030153	bacteriocin immunity	5	3	0.77	0.02901
GO:0071555	cell wall organization	145	31	22.46	0.03459
GO:0010498	proteasomal protein catabolic process	9	4	1.39	0.03764
**Cellular Components:**					
GO:0009289	pilus	37	21	6.21	3.5e-08
GO:0005576	extracellular region	224	68	37.58	7.2e-08
GO:0016021	integral component of membrane	1897	362	318.29	2.9e-06
GO:0009986	cell surface	31	12	5.2	0.0029
GO:0030430	host cell cytoplasm	14	7	2.35	0.0047
**Metabolic Functions:**					
GO:0009055	electron transfer activity	299	82	44.94	1.8e-08
GO:0016987	sigma factor activity	242	66	36.37	4.0e-07
GO:0003677	DNA binding	942	170	141.57	2.5e-05
GO:0004252	serine-type endopeptidase activity	46	16	6.91	0.00069
GO:0008233	peptidase activity	239	56	35.92	0.00539
GO:0008982	protein-N(PI)-phosphohistidine-sugar phosphotransferase activity	32	11	4.81	0.01755
GO:0090563	protein-phosphocysteine-sugar phosphotransferase activity	11	5	1.65	0.02138
GO:0008061	chitin binding	8	4	1.2	0.02142
GO:0003951	NAD+ kinase activity	8	4	1.2	0.02142
GO:0008236	serine-type peptidase activity	88	26	13.23	0.02260
GO:0016757	glycosyltransferase activity	154	30	23.14	0.02532
GO:0005355	glucose transmembrane transporter activity	6	4	0.9	0.02664

GO:0004298	threonine-type endopeptidase activity	5	3	0.75	0.02670
GO:2001070	starch binding	5	3	0.75	0.02670
GO:0052593	tryptamine:oxygen oxidoreductase (deaminating) activity	5	3	0.75	0.02670
GO:0052594	aminoacetone:oxygen oxidoreductase(deaminating) activity	5	3	0.75	0.02670
GO:0052595	aliphatic-amine oxidase activity	5	3	0.75	0.02670
GO:0052596	phenethylamine:oxygen oxidoreductase (deaminating) activity	5	3	0.75	0.02670
GO:0008131	primary amine oxidase activity	5	3	0.75	0.02670
GO:0008658	penicillin binding	14	5	2.1	0.04690

Supplementary Table 22: Significantly enriched gene ontology (GO) categories of the endophytic microbiome genes that were unique to the root metapangenome from AAA-genotype *Musa* (DC = *M. acuminata* Dwarf Cavendish) compared to BB-genotype *Musa* (BB = *M. balbisiana* 'Thai Black', MB = *M. balbisiana*) shown in **Table 1**, i.e., genes not shared with root BB and AAA+BB metapangenomes.

GO_ID	Term	Annot-ated	Signif-icant	Expected	P-value
**Biological Processes:**					
GO:0042203	toluene catabolic process	13	5	0.43	3.8e-05
GO:0009399	nitrogen fixation	34	6	1.12	0.00072
GO:0045926	negative regulation of growth	16	4	0.53	0.00151
GO:0030245	cellulose catabolic process	17	4	0.56	0.00192
GO:0045927	positive regulation of growth	12	3	0.39	0.00617
GO:0006352	DNA-templated transcription, initiation	253	16	8.32	0.00856
GO:0016075	rRNA catabolic process	6	2	0.2	0.01477
GO:0017000	antibiotic biosynthetic process	62	6	2.04	0.01551
GO:0006265	DNA topological change	7	2	0.23	0.02024
GO:0042203	toluene catabolic process	13	5	0.43	3.8e-05
GO:0009399	nitrogen fixation	34	6	1.12	0.00072
GO:0045926	negative regulation of growth	16	4	0.53	0.00151
GO:0030245	cellulose catabolic process	17	4	0.56	0.00192
GO:0045927	positive regulation of growth	12	3	0.39	0.00617
GO:0006352	DNA-templated transcription, initiation	253	16	8.32	0.00856
GO:0016075	rRNA catabolic process	6	2	0.2	0.01477
GO:0017000	antibiotic biosynthetic process	62	6	2.04	0.01551
GO:0006265	DNA topological change	7	2	0.23	0.02024
**Cellular Components:**					
GO:0005576	extracellular region	224	20	6.84	0.00011
GO:0005615	extracellular space	17	3	0.52	0.01381
GO:0033644	host cell membrane	12	2	0.37	0.04996
**Metabolic Functions:**					
GO:0051537	2 iron, 2 sulfur cluster binding	107	11	3.59	0.00086
GO:0008810	cellulase activity	15	4	0.5	0.00126
GO:0016987	sigma factor activity	242	16	8.12	0.00714
GO:0051538	3 iron, 4 sulfur cluster binding	14	3	0.47	0.01033
GO:0003690	double-stranded DNA binding	108	6	3.63	0.02788
GO:0004521	endoribonuclease activity	35	4	1.17	0.02865
GO:0004144	diacylglycerol O-acyltransferase activity	10	2	0.34	0.04226
GO:0004315	3-oxoacyl-[acyl-carrier-protein] synthase activity	24	3	0.81	0.04494

Supplementary Table 23: Significantly enriched gene ontology (GO) categories of the endophytic microbiome genes that were unique to the leaf metapangenome from BB-genotype *Musa* (BB = *M. balbisiana* 'Thai Black', MB = *M. balbisiana*) compared to AAA-genotype *Musa* (DC = *M. acuminata* Dwarf Cavendish) shown in **Table 1**, i.e., genes not shared with leaf AAA and AAA+BB metapangenomes.

GO_ID	Term	Annot-ated	Signif-icant	Expected	P-value
**Biological Processes:**					
GO:0022904	respiratory electron transport chain	87	78	73.31	1.2e-05
GO:0019439	aromatic compound catabolic process	221	201	186.22	0.013
GO:0009636	response to toxic substance	62	56	52.24	0.016
GO:0045454	cell redox homeostasis	20	20	16.85	0.032
GO:0017000	antibiotic biosynthetic process	36	35	30.33	0.038
**Cellular Components:**					
GO:0016021	integral component of membrane	1354	1153	1127.5	0.00013
GO:0005576	extracellular region	128	116	106.59	0.00742
**Metabolic Functions:**					
GO:0009055	electron transfer activity	186	164	158.01	0.00071
GO:0051537	2 iron, 2 sulfur cluster binding	75	71	63.71	0.00780
GO:0046872	metal ion binding	1151	999	977.77	0.02046
GO:0003824	catalytic activity	3380	2901	2871.31	0.02234
GO:0004519	endonuclease activity	82	75	69.66	0.03079
GO:0016491	oxidoreductase activity	971	845	824.86	0.04479
GO:0051536	iron-sulfur cluster binding	218	192	185.19	0.04592

Supplementary Table 24: Significantly enriched gene ontology (GO) categories of the endophytic microbiome genes that were unique to the leaf metapangenome from AAA-genotype *Musa* (DC = *M. acuminata* Dwarf Cavendish) compared to BB-genotype *Musa* (BB = *M. balbisiana* 'Thai Black', MB = *M. balbisiana*) shown in **Table 1**, i.e., genes not shared with leaf BB and AAA+BB metapangenomes.

GO_ID	Term	Annot-ated	Signif-icant	Expected	P-value
**Biological Processes:**					
GO:0070814	hydrogen sulfide biosynthetic process	5	1	0.02	0.0237
GO:0035435	phosphate ion transmembrane transport	6	1	0.03	0.0284
GO:0001666	response to hypoxia	6	1	0.03	0.0284
GO:0010039	response to iron ion	8	1	0.04	0.0377
GO:0006276	plasmid maintenance	8	1	0.04	0.0377
GO:0000103	sulfate assimilation	8	1	0.04	0.0377
GO:0030091	protein repair	9	1	0.04	0.0424
GO:0042773	ATP synthesis coupled electron transport	9	1	0.04	0.0424
GO:0006414	translational elongation	14	2	0.07	0.0446
**Cellular Components:**					
GO:0005618	cell wall	76	2	0.33	0.0415
**Metabolic Functions:**					
GO:0042802	identical protein binding	185	4	0.98	0.0154
GO:0070402	NADPH binding	8	1	0.04	0.0417
GO:0070404	NADH binding	8	1	0.04	0.0417

Supplementary Table 25: Significantly enriched gene ontology (GO) categories of the endophytic microbiome genes that were unique to the root metapangenome from *M. balbisiana* 'Thai Black' (BB), i.e., genes not shared with the leaf metapangenome of BB.

GO_ID	Term	Annot-ated	Signif-icant	Expected	P-value
**Biological Processes:**					
GO:0030435	sporulation resulting in formation of a cellular spore	122	35	7.54	5.6e-16
GO:0022904	respiratory electron transport chain	158	33	9.77	1.9e-10
GO:0006313	transposition, DNA-mediated	551	64	34.06	3.5e-07
GO:0030420	establishment of competence for transformation	25	9	1.55	2.9e-06
GO:0015074	DNA integration	482	53	29.8	2.0e-05
GO:0009847	spore germination	9	5	0.56	9.0e-05
GO:0030436	asexual sporulation	10	4	0.62	0.0022
GO:0019941	modification-dependent protein catabolic process	7	3	0.43	0.0068
GO:0006352	DNA-templated transcription, initiation	345	33	21.33	0.0076
GO:0010498	proteasomal protein catabolic process	9	3	0.56	0.0149
GO:0042742	defense response to bacterium	28	5	1.73	0.0266
GO:0006741	NADP biosynthetic process	5	2	0.31	0.0336
GO:0070395	lipoteichoic acid biosynthetic process	5	2	0.31	0.0336
GO:0006769	nicotinamide metabolic process	5	2	0.31	0.0336
GO:0019835	cytolysis	31	5	1.92	0.0396
**Cellular Components:**					
GO:0016021	integral component of membrane	2287	181	140.8	5.4e-09
GO:0005886	plasma membrane	1756	124	108.11	0.0017
GO:0005576	extracellular region	272	28	16.75	0.0048
GO:0031160	spore wall	11	4	0.68	0.0330
**Metabolic Functions:**					
GO:0003677	DNA binding	1635	139	91.85	2.0e-10
GO:0009055	electron transfer activity	336	44	18.88	1.1e-08
GO:0004803	transposase activity	550	64	30.9	1.1e-08
GO:0003676	nucleic acid binding	2104	175	118.2	0.00017
GO:0016987	sigma factor activity	332	33	18.65	0.00094
GO:0004252	serine-type endopeptidase activity	53	7	2.98	0.02772
GO:0008976	polyphosphate kinase activity	5	2	0.28	0.02812
GO:0017108	5'-flap endonuclease activity	5	2	0.28	0.02812

Supplementary Table 26: Significantly enriched gene ontology (GO) categories of the endophytic microbiome genes that were unique to the leaf metapangenome from *M. balbisiana* 'Thai Black' (BB), i.e., genes not shared with the root metapangenome of BB.

GO_ID	Term	Annot-ated	Signif-icant	Expected	P-value
**Biological Processes:**					
GO:0015074	DNA integration	239	22	6.81	7.6e-07
GO:0006313	transposition, DNA-mediated	244	22	6.95	1.1e-06
GO:0043709	cell adhesion involved in single-species biofilm formation	10	3	0.28	0.0023
GO:0006065	UDP-glucuronate biosynthetic process	7	2	0.2	0.0154
GO:0042026	protein refolding	7	2	0.2	0.0154
GO:0006542	glutamine biosynthetic process	10	2	0.28	0.0312
GO:0019605	butyrate metabolic process	12	2	0.34	0.0441
**Cellular Components:**					
GO:0009289	pilus	17	3	0.41	0.0071
GO:0016021	integral component of membrane	1690	49	40.46	0.0189
**Metabolic Functions:**					
GO:0004803	transposase activity	243	22	6.66	6.2e-07
GO:0003677	DNA binding	1057	46	28.96	0.00037
GO:0004494	methylmalonyl-CoA mutase activity	6	2	0.16	0.01041
GO:0008745	N-acetylmuramoyl-L-alanine amidase activity	7	2	0.19	0.01432
GO:0030246	carbohydrate binding	78	6	2.14	0.01665
GO:0042803	protein homodimerization activity	38	4	1.04	0.01945
GO:0051082	unfolded protein binding	23	3	0.63	0.02393
GO:0004497	monooxygenase activity	116	8	3.18	0.04540
GO:0106310	protein serine kinase activity	13	2	0.36	0.04776

Supplementary Table 27: Significantly enriched gene ontology (GO) categories of the endophytic microbiome genes that were unique to the root metapangenome from *M. balbisiana* (MB), i.e., genes not shared with the leaf metapangenome of MB.

GO_ID	Term	Annot-ated	Signif-icant	Expected	P-value
**Biological Processes:**					
GO:0006313	transposition, DNA-mediated	551	69	16.86	3.6e-26
GO:0015074	DNA integration	482	56	14.75	2.5e-19
GO:0032196	transposition	570	72	17.45	0.0079
GO:0008152	metabolic process	5211	189	159.5	0.0252
**Cellular Components:**					
GO:0030430	host cell cytoplasm	15	2	0.3	0.035
**Metabolic Functions:**					
GO:0004803	transposase activity	550	69	16.67	1.1e-25
GO:0003677	DNA binding	1635	96	49.56	1.0e-12
GO:0003676	nucleic acid binding	2104	125	63.78	5.4e-11
GO:0016798	hydrolase activity, acting on glycosyl bonds	256	12	7.76	0.0022
GO:2001070	starch binding	6	2	0.18	0.0127
GO:0005509	calcium ion binding	44	4	1.33	0.0434

Supplementary Table 28: Significantly enriched gene ontology (GO) categories of the endophytic microbiome genes that were unique to the leaf metapangenome from *M. balbisiana* (MB), i.e., genes not shared with the root metapangenome of MB.

GO_ID	Term	Annot-ated	Signif-icant	Expected	P-value
**Biological Processes:**					
GO:0006313	transposition, DNA-mediated	244	26	8.8	4.3e-07
GO:0030254	protein secretion by the type III secretion system	8	5	0.29	3.0e-06
GO:0015074	DNA integration	239	21	8.62	0.00012
GO:0015675	nickel cation transport	12	4	0.43	0.00064
GO:0015628	protein secretion by the type II secretion system	15	4	0.54	0.00163
GO:0017000	antibiotic biosynthetic process	48	6	1.73	0.00704
GO:0045227	capsule polysaccharide biosynthetic process	7	2	0.25	0.02408
GO:0006829	zinc ion transport	8	2	0.29	0.03136
GO:0022904	respiratory electron transport chain	97	8	3.5	0.03402
GO:0006824	cobalt ion transport	10	2	0.36	0.04808
**Cellular Components:**					
GO:0005576	extracellular region	169	21	6.66	4.9e-06
GO:0015627	type II protein secretion system complex	13	5	0.51	8.9e-05
GO:0016021	integral component of membrane	1690	83	66.65	0.0012
GO:0030430	host cell cytoplasm	11	4	0.43	0.0139
GO:0030257	type III protein secretion system complex	5	2	0.2	0.0143
GO:0043657	host cell	21	7	0.83	0.0380
**Metabolic Functions:**					
GO:0004803	transposase activity	243	26	8.58	3.2e-07
GO:0003677	DNA binding	1057	54	37.33	0.00069
GO:0008775	acetate CoA-transferase activity	8	3	0.28	0.00213
GO:0030570	pectate lyase activity	6	2	0.21	0.01696
GO:0008564	protein-exporting ATPase activity	6	2	0.21	0.01696
GO:0004252	serine-type endopeptidase activity	33	4	1.17	0.02778
GO:0016298	lipase activity	23	4	0.81	0.02984
GO:0008800	beta-lactamase activity	8	2	0.28	0.03021

Supplementary Table 29: Significantly enriched gene ontology (GO) categories of the endophytic microbiome genes that were unique to the root metapangenome from *M. textilis* (MT), i.e., genes not shared with the leaf metapangenome of MT.

GO_ID	Term	Annot-ated	Signif-icant	Expected	P-value
**Biological Processes:**					
GO:0006313	transposition, DNA-mediated	551	32	4.22	1.1e-22
GO:0015074	DNA integration	482	23	3.69	1.0e-13
GO:0019383	(+)-camphor catabolic process	5	1	0.04	0.038
**Cellular Components:**					
GO:0016021	integral component of membrane	2287	18	9.92	0.00012
**Metabolic Functions:**					
GO:0004803	transposase activity	550	32	3.9	6.9e-23
GO:0003677	DNA binding	1635	33	11.6	6.8e-10
GO:0003676	nucleic acid binding	2104	39	14.93	0.0028
GO:0004333	fumarate hydratase activity	5	1	0.04	0.0350
GO:0015643	toxic substance binding	5	1	0.04	0.0350
GO:0102483	scopolin beta-glucosidase activity	5	1	0.04	0.0350

Supplementary Table 30: Significantly enriched gene ontology (GO) categories of the endophytic microbiome genes that were unique to the leaf metapangenome from *M. textilis* (MT), i.e., genes not shared with the root metapangenome of MT.

GO_ID	Term	Annot-ated	Signif-icant	Expected	P-value
**Biological Processes:**					
GO:0006313	transposition, DNA-mediated	244	26	7.1	4.6e-09
GO:0015074	DNA integration	239	24	6.95	6.3e-08
GO:0051692	cellular oligosaccharide catabolic process	6	2	0.17	0.0117
GO:0030980	alpha-glucan catabolic process	7	2	0.2	0.0160
GO:0044179	hemolysis in other organism	8	2	0.23	0.0210
GO:0017000	antibiotic biosynthetic process	48	5	1.4	0.0297
GO:0008654	phospholipid biosynthetic process	70	6	2.04	0.0410
**Cellular Components:**					
GO:0009507	chloroplast	6	2	0.14	0.0078
**Metabolic Functions:**					
GO:0004803	transposase activity	243	26	7.2	8.4e-09
GO:0003676	nucleic acid binding	1374	59	40.69	0.00031
GO:0004556	alpha-amylase activity	13	4	0.39	0.00043
GO:0003677	DNA binding	1057	46	31.3	0.00043
GO:0103025	alpha-amylase activity (releasing maltohexaose)	8	3	0.24	0.00128
GO:0009881	photoreceptor activity	11	2	0.33	0.04025
GO:0005509	calcium ion binding	26	3	0.77	0.04031
GO:0004497	monooxygenase activity	116	7	3.44	0.04226

Supplementary Table 31: Significantly enriched gene ontology (GO) categories of the endophytic microbiome genes that were unique to the root metapangenome from *M. acuminata* FHIA-25 (FH), i.e., genes not shared with the leaf metapangenome of FH.

GO_ID	Term	Annot-ated	Signif-icant	Expected	P-value
**Biological Processes:**					
GO:0015074	DNA integration	482	18	3.91	1.8e-08
GO:0006313	transposition, DNA-mediated	551	17	4.46	8.0e-07
GO:0032259	methylation	117	4	0.95	0.015
GO:0046168	glycerol-3-phosphate catabolic process	5	1	0.04	0.040
GO:0051085	chaperone cofactor-dependent protein refolding	5	1	0.04	0.040
GO:0015074	DNA integration	482	18	3.91	1.8e-08
GO:0006313	transposition, DNA-mediated	551	17	4.46	8.0e-07
GO:0032259	methylation	117	4	0.95	0.015
GO:0046168	glycerol-3-phosphate catabolic process	5	1	0.04	0.040
GO:0051085	chaperone cofactor-dependent protein refolding	5	1	0.04	0.040
**Cellular Components:**					
GO:0009289	pilus	44	2	0.29	0.033
GO:0009331	glycerol-3-phosphate dehydrogenase complex	6	1	0.04	0.038
**Metabolic Functions:**					
GO:0004803	transposase activity	550	17	4.76	3.1e-06
GO:0003676	nucleic acid binding	2104	31	18.22	0.00014
GO:0003677	DNA binding	1635	21	14.16	0.02396

Supplementary Table 32: Significantly enriched gene ontology (GO) categories of the endophytic microbiome genes that were unique to the leaf metapangenome from *M. acuminata* FHIA-25 (FH), i.e., genes not shared with the root metapangenome of FH.

GO_ID	Term	Annot-ated	Signif-icant	Expected	P-value
**Biological Processes:**					
GO:0006313	transposition, DNA-mediated	244	33	6.22	4.1e-16
GO:0015074	DNA integration	239	30	6.09	9.0e-14
GO:0030163	protein catabolic process	24	3	0.61	0.0357
**Cellular Components:**					
GO:0016021	integral component of membrane	1690	38	28.09	0.0027
GO:0005615	extracellular space	16	2	0.27	0.0281
**Metabolic Functions:**					
GO:0004803	transposase activity	243	33	5.73	6.3e-17
GO:0003677	DNA binding	1057	48	24.94	2.5e-07
GO:0003676	nucleic acid binding	1374	60	32.42	5.9e-05
GO:0052874	FMN reductase (NADH) activity	5	2	0.12	0.0053
GO:0008239	dipeptidyl-peptidase activity	6	2	0.14	0.0078
GO:0008199	ferric iron binding	15	2	0.35	0.0475

Supplementary Table 33: Significantly enriched gene ontology (GO) categories of the endophytic microbiome genes that were unique to the root metapangenome from *M. acuminata* Dwarf Cavendish (DC), i.e., genes not shared with the leaf metapangenome of DC.

GO_ID	Term	Annot-ated	Signif-icant	Expected	P-value
**Biological Processes:**					
GO:0006313	transposition, DNA-mediated	551	61	19.18	5.1e-17
GO:0015074	DNA integration	482	47	16.78	4.3e-11
GO:0009399	nitrogen fixation	37	5	1.29	0.0086
GO:0042203	toluene catabolic process	13	3	0.45	0.0092
GO:0017000	antibiotic biosynthetic process	72	7	2.51	0.0122
GO:0006265	DNA topological change	7	2	0.24	0.0226
GO:0044571	[2Fe-2S] cluster assembly	7	2	0.24	0.0226
GO:0030153	bacteriocin immunity	10	2	0.35	0.0452
**Cellular Components:**					
GO:0005576	extracellular region	272	24	7.08	2.2e-07
GO:0033644	host cell membrane	13	2	0.34	0.043
**Metabolic Functions:**					
GO:0004803	transposase activity	550	61	18.86	5.9e-17
GO:0003677	DNA binding	1635	93	56.05	8.4e-09
GO:0003676	nucleic acid binding	2104	121	72.13	0.00016
GO:0051538	3 iron, 4 sulfur cluster binding	15	3	0.51	0.01336
GO:0031177	phosphopantetheine binding	29	4	0.99	0.01635
GO:0051536	iron-sulfur cluster binding	340	16	11.66	0.01962
GO:0003690	double-stranded DNA binding	109	4	3.74	0.03420
GO:0031071	cysteine desulfurase activity	10	2	0.34	0.04395

Supplementary Table 34: Significantly enriched gene ontology (GO) categories of the endophytic microbiome genes that were unique to the leaf metapangenome from *M. acuminata* Dwarf Cavendish (DC), i.e., genes not shared with the root metapangenome of DC.

GO_ID	Term	Annot-ated	Signif-icant	Expected	P-value
**Biological Processes:**					
GO:0070814	hydrogen sulfide biosynthetic process	5	1	0.01	0.0060
GO:0006048	UDP-N-acetylglucosamine biosynthetic process	5	1	0.01	0.0060
GO:0000103	sulfate assimilation	8	1	0.01	0.0095
GO:0042773	ATP synthesis coupled electron transport	9	1	0.01	0.0107
**Cellular Components:**					
**Metabolic Functions:**					
GO:0031177	phosphopantetheine binding	19	1	0.03	0.0326
GO:0008137	NADH dehydrogenase (ubiquinone) activity	24	1	0.04	0.0411

Supplementary Table 35: Significantly enriched gene ontology (GO) categories of the endophytic microbiome genes that were unique to the root metapangenome from *M. sikkimensis* (MS), i.e., genes not shared with the leaf metapangenome of MS.

GO_ID	Term	Annot-ated	Signif-icant	Expected	P-value
**Biological Processes:**					
GO:0006313	transposition, DNA-mediated	551	21	7.44	9.4e-06
GO:0019684	photosynthesis, light reaction	5	3	0.07	2.3e-05
GO:0015979	photosynthesis	38	11	0.51	2.8e-05
GO:0018298	protein-chromophore linkage	15	3	0.2	0.00096
GO:0015995	chlorophyll biosynthetic process	16	6	0.22	0.00097
GO:0030494	bacteriochlorophyll biosynthetic process	12	4	0.16	0.00168
GO:0019685	photosynthesis, dark reaction	18	4	0.24	0.00169
GO:0036070	light-independent bacteriochlorophyll biosynthetic process	7	2	0.09	0.00362
GO:0019441	tryptophan catabolic process to kynurenine	8	2	0.11	0.00479
GO:0015074	DNA integration	482	14	6.51	0.00488
GO:0019253	reductive pentose-phosphate cycle	13	2	0.18	0.01277
**Cellular Components:**					
GO:0030076	light-harvesting complex	11	9	0.12	2.9e-11
GO:0019866	organelle inner membrane	7	3	0.08	4.6e-05
**Metabolic Functions:**					
GO:0004803	transposase activity	550	21	7.41	1.1e-05
GO:0004568	chitinase activity	5	2	0.07	0.0018
GO:0004061	arylformamidase activity	5	2	0.07	0.0018
GO:0046872	metal ion binding	1762	37	23.74	0.0019
GO:0016636	oxidoreductase activity, acting on the CH-CH group of donors, iron-sulfur protein as acceptor	9	3	0.12	0.0036
GO:0016984	ribulose-bisphosphate carboxylase activity	9	2	0.12	0.0061
GO:0016730	oxidoreductase activity, acting on iron-sulfur proteins as donors	32	3	0.43	0.0089
GO:0051539	4 iron, 4 sulfur cluster binding	179	6	2.41	0.0336
GO:0030145	manganese ion binding	53	3	0.71	0.0343
GO:0003677	DNA binding	1635	30	22.03	0.0405

Supplementary Table 36: Significantly enriched gene ontology (GO) categories of the endophytic microbiome genes that were unique to the leaf metapangenome from *M. sikkimensis* (MS), i.e., genes not shared with the root metapangenome of MS.

GO_ID	Term	Annot-ated	Signif-icant	Expected	P-value
**Biological Processes:**					
GO:0015979	photosynthesis	28	9	0.81	1.2e-06
GO:0015074	DNA integration	239	18	6.95	0.00017
GO:0036070	light-independent bacteriochlorophyll biosynthetic process	5	3	0.15	0.00023
GO:0015977	carbon fixation	14	4	0.41	0.00044
GO:0015995	chlorophyll biosynthetic process	13	7	0.38	0.00232
GO:0019439	aromatic compound catabolic process	275	22	8	0.00316
GO:0018298	protein-chromophore linkage	14	3	0.41	0.00693
GO:0030494	bacteriochlorophyll biosynthetic process	10	5	0.29	0.00763
GO:0006558	L-phenylalanine metabolic process	14	3	0.41	0.00785
GO:0000917	division septum assembly	22	3	0.64	0.02474
GO:0042203	toluene catabolic process	9	2	0.26	0.02644
GO:0030245	cellulose catabolic process	10	2	0.29	0.03243
GO:0019509	L-methionine salvage from methylthioadenosine	12	2	0.35	0.04579
**Cellular Components:**					
**Metabolic Functions:**					
GO:0051537	2 iron, 2 sulfur cluster binding	100	13	3.15	1.3e-05
GO:0016984	ribulose-bisphosphate carboxylase activity	8	4	0.25	6.1e-05
GO:0031419	cobalamin binding	16	4	0.5	0.00129
GO:0004497	monooxygenase activity	116	13	3.66	0.00588
GO:0047125	delta1-piperideine-2-carboxylate reductase activity	5	2	0.16	0.00928
GO:0052595	aliphatic-amine oxidase activity	5	2	0.16	0.00928
GO:0008131	primary amine oxidase activity	5	2	0.16	0.00928
GO:0004494	methylmalonyl-CoA mutase activity	6	2	0.19	0.01364
GO:0003676	nucleic acid binding	1374	39	43.3	0.01455
GO:0004300	enoyl-CoA hydratase activity	9	2	0.28	0.03075
GO:0016705	oxidoreductase activity, acting on paired donors, with incorporation or reduction of molecular oxygen	126	13	3.97	0.04260
GO:0016730	oxidoreductase activity, acting on iron-sulfur proteins as donors	28	3	0.88	0.04489
GO:0009881	photoreceptor activity	11	2	0.35	0.04508
GO:0046872	metal ion binding	1417	56	44.66	0.04699

Supplementary Table 37: List of taxon matches and gene matches for which there was a signature of phylosymbiosis (see additional excel file) from for endophytic microbiomes of *Musa* samples, showing gene and taxon lists, counts, and complete DIAMOND blastx output.

Supplementary Figure 8: Associations between *Musa* genotypes (left) and dominant endophytic microbiome strains that displayed signatures of phylosymbiosis with their hosts (center). Size of block indicates relative abundance. Host *Musa* genotypes correspond to those listed in **Table 1** (BB = *M. balbisiana* 'Thai Black', DC = *M. acuminata* Dwarf Cavendish, FH = *M. acuminata* (FHIA-25), MB = *M. balbisiana*, MS = *M. sikkimensis*, MT = *M. textilis*). For display purposes, microbes with fewer than 18 occurrences in the gene clusters were omitted.

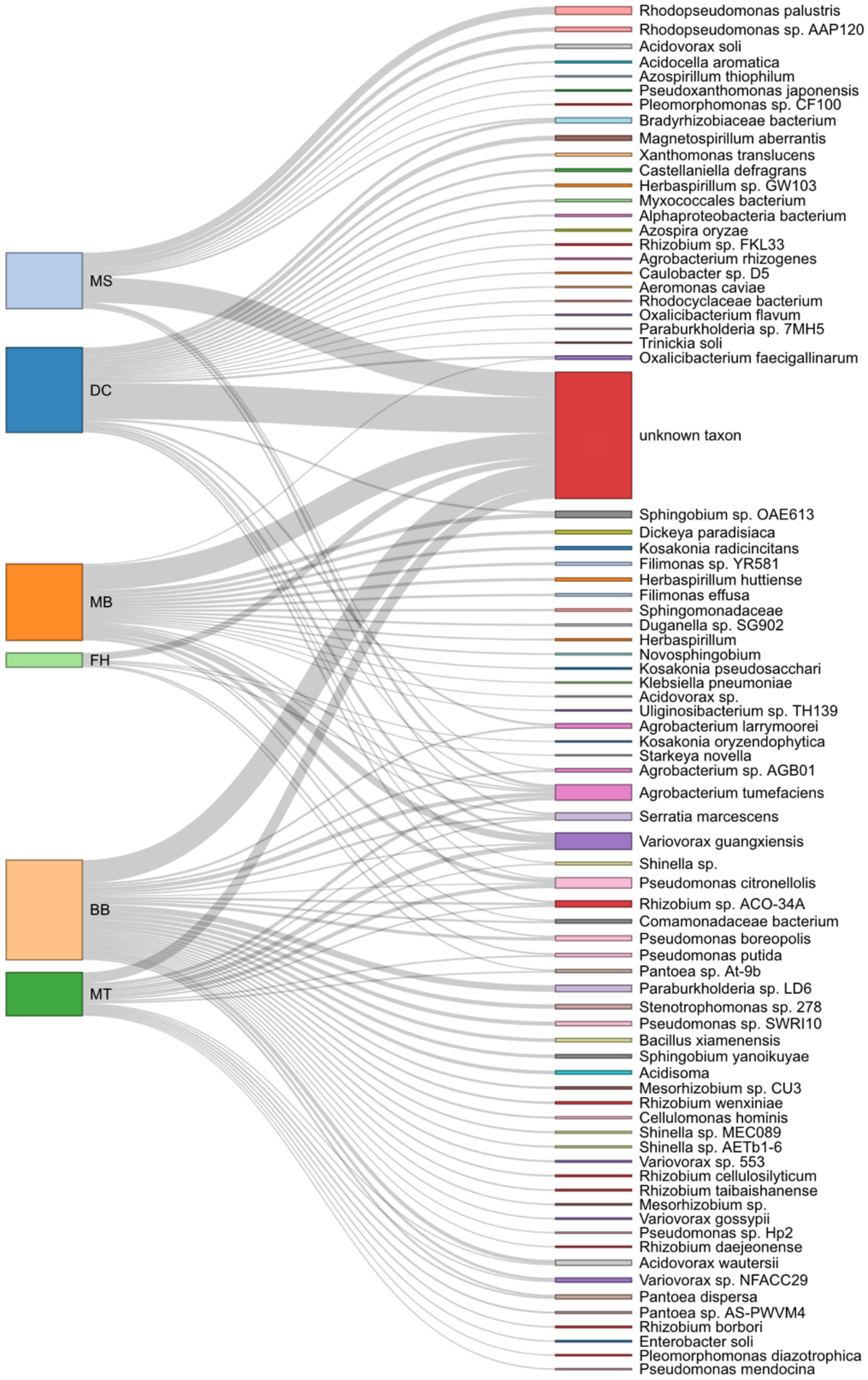

Supplementary Table 38: Significantly enriched gene ontology (GO) categories of the endophytic microbiome genes that showed signatures of phylosymbiosis with host genotype from all tissues from 12 sampled *Musa* listed in **Table 1** (*M. balbisiana* 'Thai Black', *M. acuminata* Dwarf Cavendish, *M. acuminata* (FHIA-25), *M. balbisiana*, *M. sikkimensis*, *M. textilis*).

GO_ID	Term	Annot-ated	Signif-icant	Expected	P-value
**Biological Processes:**					
GO:0006351	transcription, DNA-templated	757	13	15.36	0.0035
GO:0001123	transcription initiation from bacterial-type RNA polymerase promoter	5	2	0.1	0.0039
GO:0006614	SRP-dependent cotranslational protein targeting to membrane	5	2	0.1	0.0039
GO:0006412	translation	159	12	3.23	0.0047
GO:0017038	protein import	6	2	0.12	0.0058
GO:0042026	protein refolding	7	2	0.14	0.008
GO:0019430	removal of superoxide radicals	8	2	0.16	0.0106
GO:0019619	3,4-dihydroxybenzoate catabolic process	8	2	0.16	0.0106
GO:0006099	tricarboxylic acid cycle	43	4	0.87	0.0108
GO:0019288	isopentenyl diphosphate biosynthetic process, methylerythritol 4-phosphate pathway	10	2	0.2	0.0165
GO:0043213	bacteriocin transport	11	2	0.22	0.0199
GO:0006541	glutamine metabolic process	41	4	0.83	0.02
GO:0017004	cytochrome complex assembly	16	2	0.32	0.0407
GO:0006094	gluconeogenesis	16	2	0.32	0.0407
GO:0006189	'de novo' IMP biosynthetic process	16	2	0.32	0.0407
GO:0006353	DNA-templated transcription, termination	17	2	0.34	0.0455
**Cellular Components:**					
GO:0005829	cytosol	443	23	9.8	0.0032
GO:0005737	cytoplasm	1417	52	31.33	0.0127
GO:0022625	cytosolic large ribosomal subunit	23	3	0.51	0.0135
GO:0005886	plasma membrane	1763	50	38.98	0.0331
**Metabolic Functions:**					
GO:0005524	ATP binding	803	36	16.11	2.70E-06
GO:0003924	GTPase activity	38	6	0.76	9.70E-05
GO:0005525	GTP binding	79	8	1.59	0.00017
GO:0003735	structural constituent of ribosome	68	6	1.36	0.00234
GO:0046872	metal ion binding	1789	47	35.89	0.00242
GO:0003899	DNA-directed 5'-3' RNA polymerase activity	14	3	0.28	0.00245
GO:0051539	4 iron, 4 sulfur cluster binding	180	10	3.61	0.0033
GO:0008948	oxaloacetate decarboxylase activity	9	2	0.18	0.01313
GO:0046933	proton-transporting ATP synthase activity, rotational mechanism	10	2	0.2	0.0162
GO:0004176	ATP-dependent peptidase activity	11	2	0.22	0.01954
GO:0042626	ATPase-coupled transmembrane transporter activity	183	14	3.67	0.02094
GO:0019843	rRNA binding	58	5	1.16	0.02232
GO:0043022	ribosome binding	14	2	0.28	0.03109
GO:0051538	3 iron, 4 sulfur cluster binding	16	2	0.32	0.03994
GO:0003746	translation elongation factor activity	16	2	0.32	0.03994
